# The association between diabetes mellitus and prostate cancer: a meta-analysis and Mendelian randomization

**DOI:** 10.18632/aging.205886

**Published:** 2024-06-04

**Authors:** Gui-Chen Ye, Yu-Xuan Yang, Kuang-Di Luo, Shao-Gang Wang, Qi-Dong Xia

**Affiliations:** 1 Department and Institute of Urology, Tongji Hospital, Tongji Medical College, Huazhong University of Science and Technology, Wuhan 430030, China

**Keywords:** meta-analysis, diabetes mellitus, prostate cancer, Mendelian randomization, obesity

## Abstract

Background: Prostate cancer is one of the most common types of cancer in the US, and it has a high mortality rate. Diabetes mellitus is also a dangerous health condition. While some studies have examined the relationship between diabetes mellitus and the risk of prostate cancer, there is still some debate on the matter. This study aims to carefully assess the relationship between prostate cancer and diabetes from both real-world and genetic-level data.

Methods: This meta-analysis was conducted following the PRISMA 2020 reporting guidelines. The study searched three databases including Medline, Embase and Cochrane. The studies about the incidence risk of prostate cancer with diabetes mellitus were included and used to evaluate the association. The odds ratio (OR), risk ratio (RR) and 95% confidence intervals (95% CI) were estimated using Random Effects models and Fixed Effects models. Mendelian randomization study using genetic variants was also conducted.

Results: A total of 72 articles were included in this study. The results showed that risk of prostate cancer decreased in diabetes patients. And the influence was different in different regions. This study also estimated the impact of body mass index (BMI) in the diabetes populations and found that the risk decreased in higher BMI populations. The MR analysis found that diabetes mellitus exposure reduced the risk of prostate cancer in the European population and Asia populations.

Conclusions The diabetes mellitus has a protective effect on prostate cancer. And the influence of obesity in diabetes mellitus plays an important role in this effect.

## INTRODUCTION

Nowadays, cancer has become a tremendous burden on people’s health and society’s economy. For males, prostate cancer is one of the most important diseases, and people are paying more attention to it. As reported, prostate cancer is the most common cancer in the US, and the mortality is the second, just behind cancer of the lung [1]. Besides, even in Asia, a traditional low-incidence risk region, the risk of prostate cancer is rapidly increasing [2]. Thus, prostate cancer has become an emergency challenge to the health of human beings.

Prostate cancer is a kind of cancer that occurs in the prostate, most of which are prostatic intraepithelial neoplastic [3]. Obesity plays an essential role in occurence of prostate cancer. The mortality and the risk of recurrence increased in obese patients, leading to a worse prognosis [4]. The incidence risk of prostate cancer is also associated with age, family history, and physical activity [5].

Diabetes mellitus, a kind of metabolic syndrome with chronic hyperglycemia [6], is also a dangerous factor to human health, especially in developed countries. In 2017 there were about 425 million cases, which is projected to rise to 693 million by 2045 [7]. In recent years, the association between diabetes mellitus and the risk of cancer has gotten researchers’ attention, and several studies confirm that there is a strong association between diabetes and cancers. [8–10] The neoplastic cell can be activated by the IGF-1 or IGF-2 and hyperglycemia also plays a vital role in the growth of neoplasm [8]. But as for prostate cancer, there are some different opinions. Some studies found that the incidence risk decreased in diabetes mellitus patients [10, 11], while others have an opposite conclusion [12, 13]. This phenomenon is confusing and the detailed mechanism still needs further study. The study of the association between diabetes mellitus and the risk of prostate cancer can help us understand and manage the disease better.

With the development of bioinformatics, we now have more tools to study the association between diabetes mellitus and the risk of prostate cancer. Mendelian randomization (MR) is a new tool to evaluate the association between the causal effects using genetic variants. It uses genome-wide association studies (GWASs) to assess the result which can avoid both the researching bias and confounding factors [14]. In this study, we first conducted a meta-analysis using data from the real-world study. Then, MR was applied to evaluate and confirm the result of meta-analysis. The results may offer a new vision of the relationship between prostate cancer and diabetes mellitus.

## MATERIALS AND METHODS

### Selection criteria

The inclusion and exclusion criteria were as follows:

1. The exposure we were interested in was suffering from diabetes mellitus
2. The outcome of interest was suffering from prostate cancer
3. This study only included the latest datasets reported in different articles.
4. The patients with secondary tumors of prostate cancer were excluded.
5. Studies using cell or animal models and case reports were excluded.
6. Studies without full text or lack of data were excluded. The study language was limited to English.

### Search strategy

**Figure 1:**
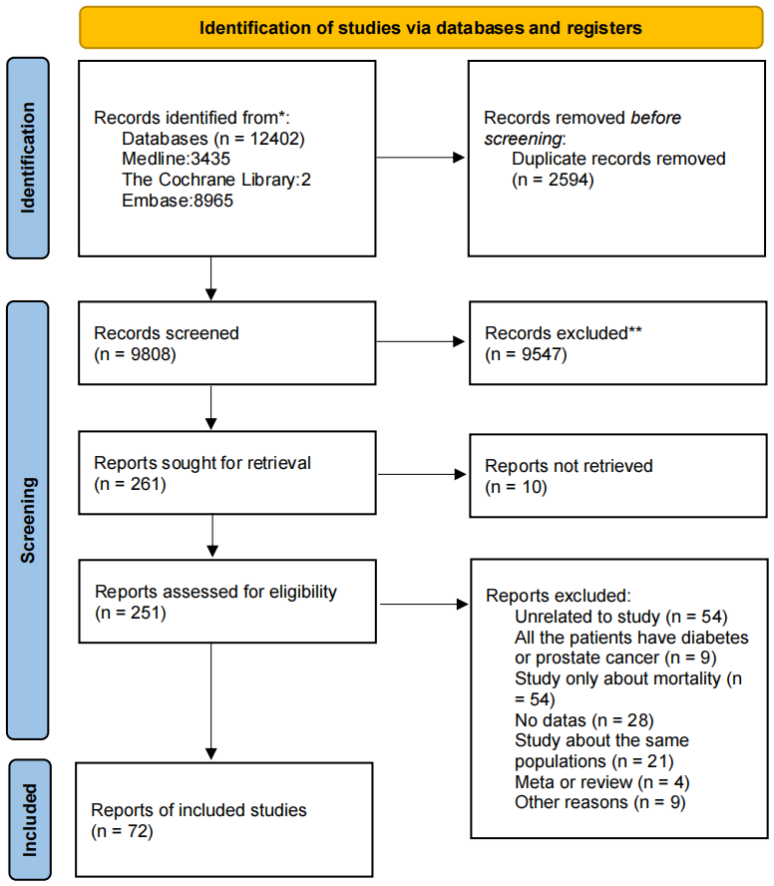
PRISMA (Preferred Reporting Items for Systematic Reviews and Meta-Analyses) flowchart for study selection for the systematic review on diabetes mellitus and the risk of prostate cancer.

This study was conducted according to the Preferred Reporting Items for Systematic Reviews and Meta-analyses (PRISMA) 2020 reporting guideline. We searched PubMed, Cochrane, and EMBASE to evaluate the association between diabetes mellitus and the risk of prostate cancer from inception to September 17, 2023. We also used Google Scholar to get gray literature, just like conference abstracts. The searching keywords were “Diabetes mellitus”, “Prostate cancer” and “Prostate neoplasms”. The detailed search strategy for each database was offered in Supporting Information: Supplementary Table 1. Two reviewers, Y.G.C and Y.Y.X, searched for the title and abstract independently, and the discrepancies of including were judged by an independent third authors X.Q.D. The Endnote application (20 version) was used to remove duplicate articles and literate the articles. A PRISMA flow chart in Figure 1 was used to depict the literature search procedure. This systematic review and meta-analysis study was registered in PROSPERO (CRD42023461982).

### Data extraction

We used a designed data extraction sheet to extract information from the included studies. The data extraction sheet consists of the article’s name, author’s name, publication’s year, study type, region, sample source, matching criteria, treatment, and type of diabetes mellitus. The background information, like the age of patients, was also included. The detailed characteristics of the articles included in this study are shown in Supplementary Table 9. The outcomes of interest were the risk of prostate cancer.

### Literature quality assessment

The Newcastle–Ottawa scale (NOS) was used to evaluate the quality of case-control studies and cohort studies. The evaluation criteria for an observational study of the Agency for Healthcare Research and Quality (AHRQ) were used to evaluate the quality of cross-sectional studies.

The NOS mainly considers three methods: selection, comparability, and outcome. And it has eight points. A study can score one in the items in selection and outcome, while the score in comparability is from zero to two. If the score is under 4, it will be considered as a bad quality study. The AHRQ criteria consist of eleven points, and the answer can be ‘yes’, ‘no’ and ‘unknown’. If the answer is ‘yes’, the item scores one, or it scores zero. The scores between 8 and 11 are regarded as good quality, 4 and 6 as moderate quality, and 0 and 3 as bad quality.

Two researchers, Y.G.C and Y.Y.X, evaluated the quality and the bias of studies independently and the discrepancies were judged by the third researcher, X.Q.D.

### Data synthesis and statistical analysis

This study estimated the association between prostate cancer and diabetes mellitus using Random Effects (RE) models or Fixed Effects (FE) models. We extracted the odds ratio (OR), risk ratio (RR), and their 95% CI of adjusted DM, which could provide sufficient data. Besides, we also calculated the unadjusted OR, RR, and 95% CI of DM by using the data from included articles. The pooled risk ratio (pRR) and pooled odds ratio(pOR) were calculated with 95% CI. The heterogeneity between studies was also analyzed using the standard Cochrane Chi-square χ^2^(Cochrane’s Q) test and the I^2^ test. Sensitivity analyses were conducted if I^2^>50% or α > 0.10. All the p-values were two-sided, and a p<0.05 was considered significantly different. All the analyses were performed in R software (version 4.2.1) with the “meta” package.

### MR

The data resources were obtained from MRC IEU OpenGWAS (https://gwas.mrcieu.ac.uk/; version: v7.5.12 - 2023-09-27), developed at the MRC Integrative Epidemiology Unit at the University of Bristol. The GWAS ID of the included study were: bbj-a-153 (Type 2 diabetes), bbj-a-148 (Prostate cancer), ebi-a-GCST007517 (Type 2 diabetes), ebi-a-GCST007516 (Type 2 diabetes (adjusted for BMI), ebi-a-GCST90018905 (Prostate cancer). The detailed information was shown in Supporting Information: Supplementary Table 2. All the p-values were two-tailed. The R software (version 4.2.1) with the ‘TwoSampleMR’ package was used for the analysis.

### Availability of data and materials

All the articles and GWAS data are available on public dataset. The datasets generated during and/or analyzed during the current study are available from the corresponding author on reasonable request.

## RESULTS

This study sought 261 reports for retrieval from the database or other sources. After using inclusion and exclusion tools, 189 articles were excluded, and 72 were included in this meta-analysis study. Of the 188 articles, 55 were found unrelative to our study after reading the full text. 54 articles only reported the mortality. 21 articles reported the same populations, 28 with no complete data, 4 were reviews of meta-analysis, 9 were studies for patients, and 9 for other reasons.

### Characteristics of included studies and patients

The characteristics of the 72 articles included in this study were shown in Supplementary Table 9. Among the studies, 35 were case-control studies, 31 were cohort studies, and 6 were cross-section studies. The match population in the case-control was varied, such as the patients in the same hospital and those in the same area. The total number of the population was 1,268,481. The regions of the studies were all over the world, such as Asia, North America, European, Africa, and Oceania. In the studies, most of the population was over 40 years old, and the average age was about 60. Most patients were identified from the national cancer registry or local medical records. 29 studies reported the influence of BMI, and 20 studies also reported the treatments of the patients.

### Quality assessment of the included studies

This study used the Agency for Healthcare Research and Quality (AHRQ) criteria to evaluate cross-sectional studies, and the Newcastle–Ottawa scale (NOS) was used to assess the quality of case-control studies and cohort studies. The detailed results were shown in the Supporting Information: Supplementary Tables 3–5. The incuded 71 articles were evaluated as high or moderate quality. Only 1 meeting abstract had little information about the study, but we still included this article in our study.

### The association between diabetes mellitus and prostate cancer risk

**Figure 2:**
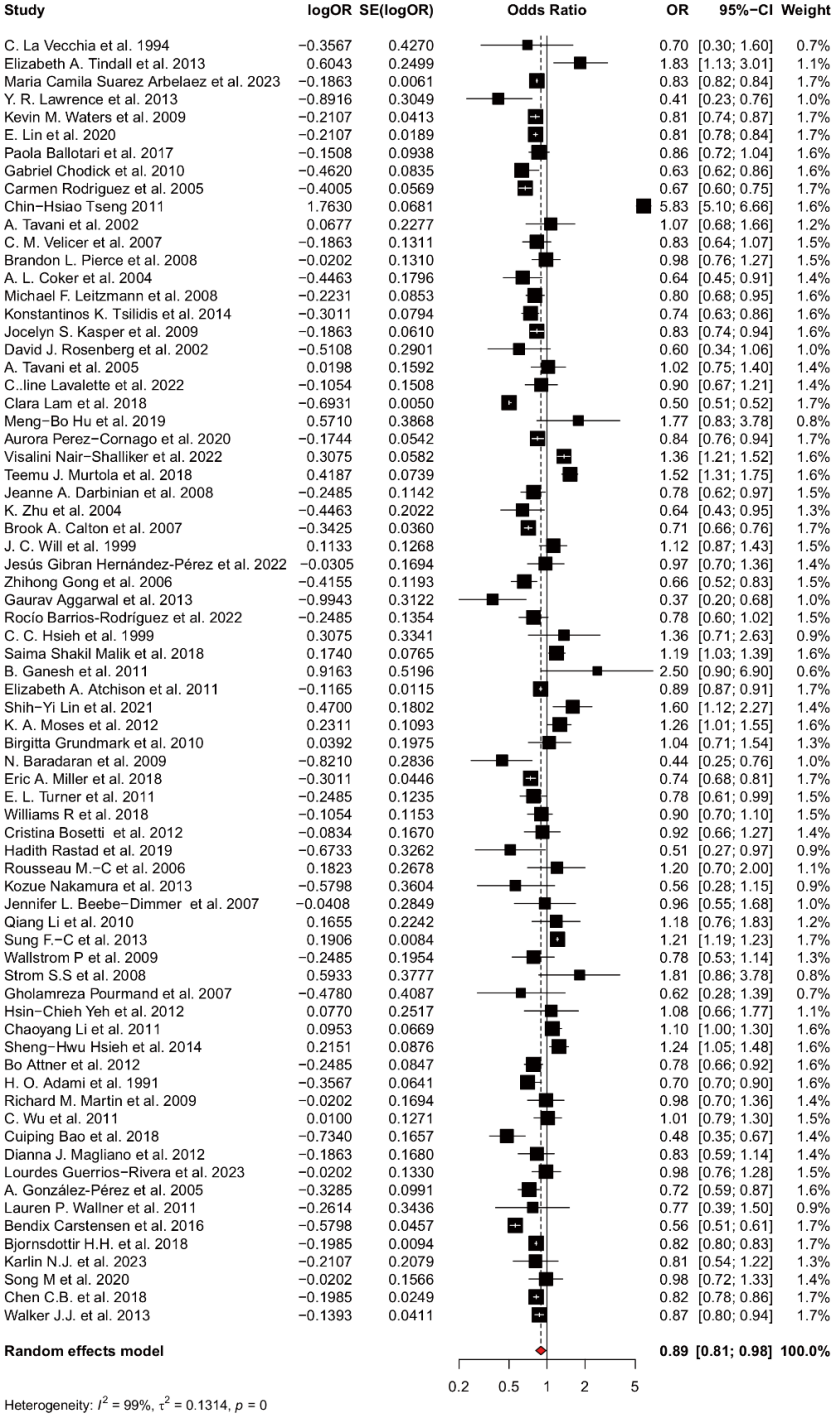
**Forest plot for the association between suffering diabetes mellitus and the risk of prostate cancer.** Pooled prevalence and 95% confidence intervals of prostate cancer risk associated with diabetes mellitus.

**Figure 3:**
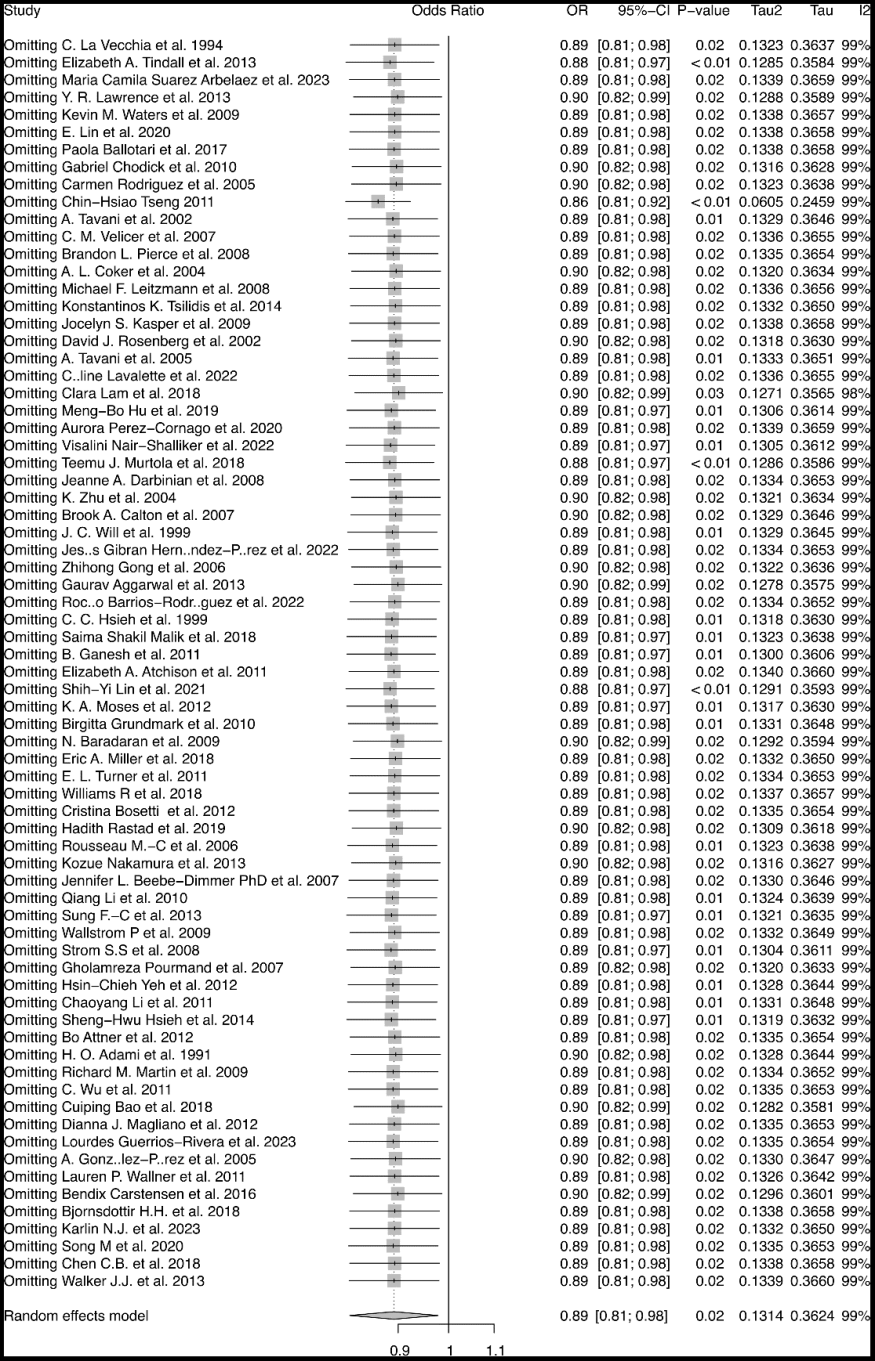
**Sensitivity analysis of the association between diabetes mellitus and risk of prostate cancer.** Sensitivity analysis by stepwise omitting the included studies.

A total of 1 268 481 people were included in this study. The result of the meta-analysis was shown in Figure 2. There was statistical significance in the connection between the risk of prostate cancer and diabetes mellitus, with a pOR of 0.89 (95% CI: 0.82-0.98, I2 = 99%, RE model). Notably, there is a study in Asia with significant heterogeneity on the study [12]. After excluding this study, the pOR changed to 0.86 (95% CI: 0.81-0.92, I2 = 99%, RE model. Figure 3). This means the incidence risk of prostate cancer significantly decreases in patients with diabetes mellitus.

**Figure 4:**
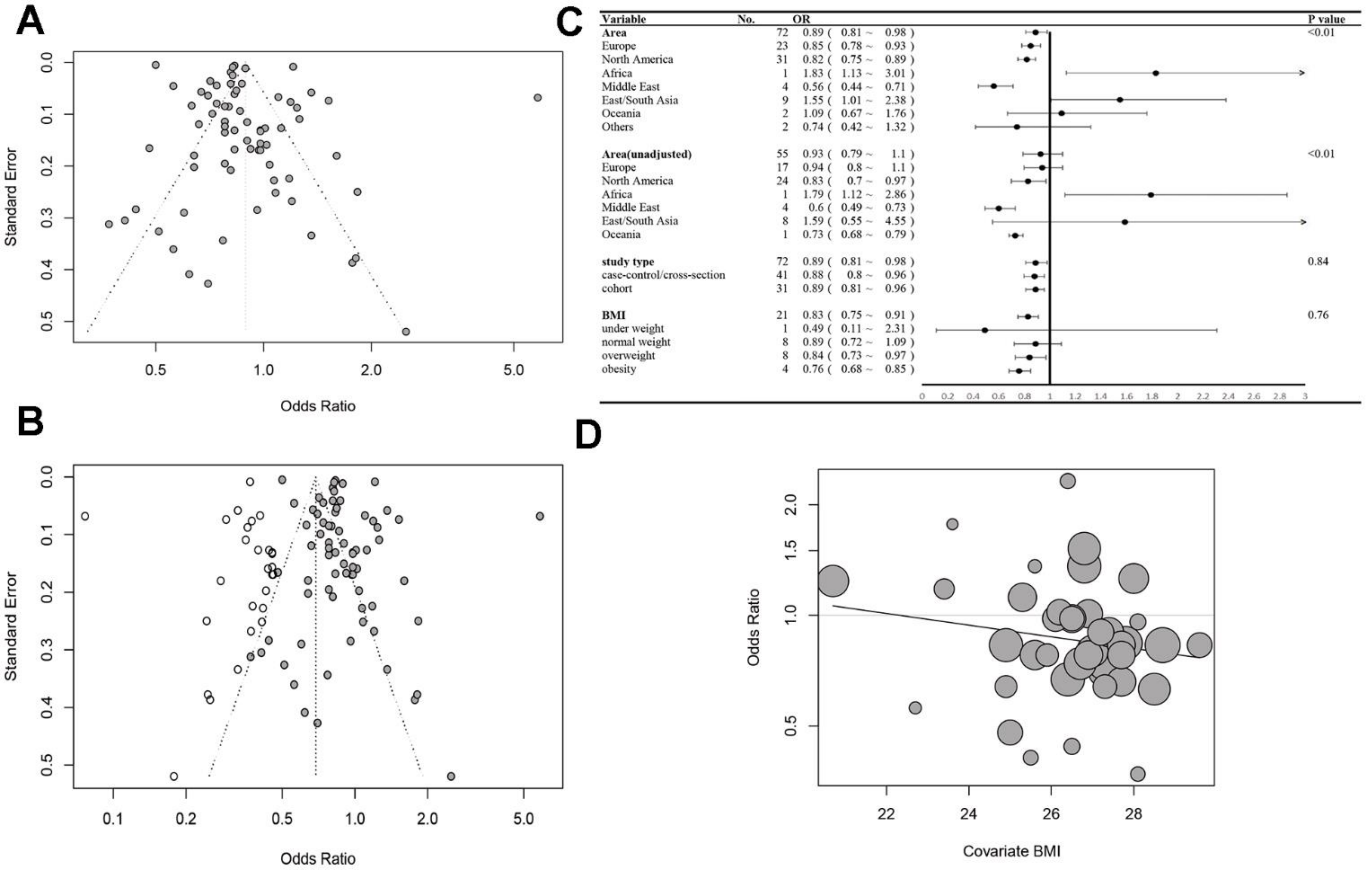
(**A**) Funnel plot of the meta-analysis. (**B**) The trim and fill funnel plot. (**C**) Subgroup analyses for OR of prostate cancer progression stratified by area, study type and BMI. (**D**) The meta-regression for OR of PCa and BMI.

Following this, a subgroup analysis stratified by region was also performed. In the study, we found out that in European, North America, and Middle East, the incidence risk of prostate cancer decreased in diabetes mellitus patients, while in the East Asia the incidence risk increased (Figure 4C). Interestingly, the results were almost on the contract. Moreover, in the subgroup analysis of unadjusted effects, we found that there was no significant pOR in European populations.

This funnel plot was shown in Figure 4A. We found it did not show good symmetry, so the trim and fill method was used to eliminate the latent publication (Figure 4B). It indicated that the publication bias had weakened the effect of diabetes on the incidence of PCa. The cumulative meta-analysis showed that a study in Asia played an important role in the result, and the incidence risk of prostate cancer decreased significantly after it was excluded. Besides, the study did identify a statistically significant publication bias based on Begg’s test (z = -3.01, p-value = 0.0026) and Egger’s test (t = 1.75, df = 70, p-value = 0.0847).

### The association between BMI and risk of prostate cancer in diabetes mellitus patients

In this study, we found 8 articles with stratification analysis of body mass index. We defined the BMI under 18.5 as underweight, 18.5~24.9 as normal weight, 25~29.9 as overweight, and more than 30 as obesity. We found that the influence of diabetes mellitus increased in the population with higher BMI. The results showed a pRR of 0.83 (95% CI:0.75~0.91, I2 = 44%, FE model, Figure 4C). It showed that there was no significant association in the normal weight population (pRR=0.89, 95% CI: 0.72~1.09). And the risk decreases in overweight people (pRR=0.84, 95% CI: 0.73~0.97) and obesity (pRR=0.76, 95% CI :0.68~0.85).

Thus, a meta-regression was also conducted with 40 studies offering the BMI data of the population to investigate the association between BMI and publication OR/ RR. We estimated overall BMI by the percentage of people in different segments and used the medium BMI of every segment as the average BMI. The results showed a negative tendency between BMI and risk of prostate cancer (p=0.207, Figure 4D), though the result was not precise, and more studies had to be conducted.

### MR

The data source for this two-sample MR study was shown in Supporting Information Supplementary Table 2. The results of MR in European and East Asian populations were shown in the Supporting Information: Supplementary Tables 6–8. And pleiotropy and heterogeneity tests were also included.

**Figure 5:**
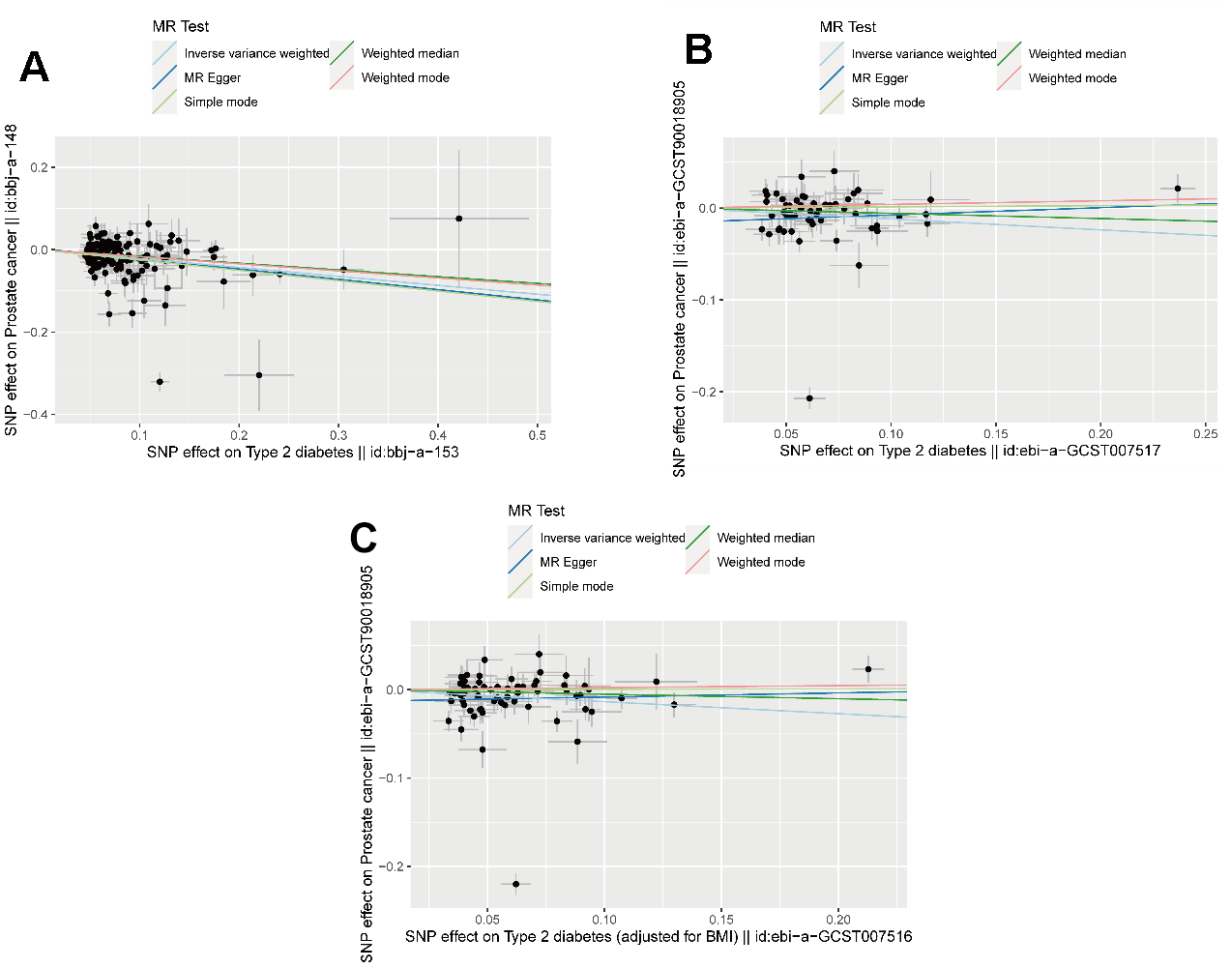
**The result of Mendelian randomization.** (**A**) The scatter plot of MR in East Asia population (**B**) The scatter plot of MR in European population (**C**) The scatter plot of MR in European population adjusted by BMI.

As Figure 5 showed, we analyzed and found out that in East Asian populations, the risk of prostate cancer decreased in the people suffering from diabetes mellitus (β=-0.21652, p<0.01, Figure 5A), which is opposite to the result of the meta-analysis. In the European populations, the risk decreased in diabetes mellitus after adjusted by BMI (β=-0.13575, p=0.04342, Figure 5C). The forest plots by Leave-One-Out were offered in Supplementary Figure 1.

## DISCUSSION

In recent years, some review and meta-analysis studies reported the association between diabetes mellitus and the risk of prostate cancer, but there still are some problems to solve. In the post studies, the overall risk of prostate cancer was decreased in diabetes mellitus patients except in the studies conducted in Asia. [11, 13, 15–18]. So, we made this meta-analysis and MR analysis to reevaluate the association between diabetes mellitus and the risk of prostate cancer. A total of 35 case-control studies, 31 cohort studies, and 6 cross-section studies were included. The total of 1,268,481 people came from different regions such as the US, UK, China, Japan, Israel, Sweden, and so on. We found that in North America, the Middle East, and Europe, diabetes mellitus was a protective factor to prostate cancer, while the result in East Asia was on the contrary. This result was consistent with some previous meta-analysis [13, 15, 19–21]. As for unmeasured confounding meta-analysis, we had a different conclusion from the study conducted in 2020 [22]. We thought there was not enough evidence to confirm the association between diabetes mellitus and prostate cancer (Figure 4C), because that meta-analysis didn’t include enough studies conducted in Asia [22].

Actually, this protective effect’s mechanism is still debated and not fully delineated. The low level of testosterone and hypoinsulinemia can be possible reasons [8]. The insulin-like growth factor (IGF) plays an important role in growth and also participates in the development of pathological situations, especially tumorigenesis [23]. Insulin can also be affected through IGF receptor (IGFR) [24]. IGFR was found to be overexpressing in prostate cancer, and the inhibition of IGF-1 receptor has a therapeutic efficiency in preclinical studies [25]. Hypoinsulinemia in Type 1 diabetes mellitus(T1DM) and long-lasting Type 2 diabetes mellitus(T2DM) may cause protective effects. Some studies also reported low testosterone in diabetes mellitus patients [26, 27], though the protective effect is controversial [28].

It is generally believed that diabetes mellitus is associated with decreased incidence of prostate cancer. But in subgroup analysis, we found the effect of diabetes mellitus was not the same in different regions, especially in East Asia. We first thought about the different expressions on genetic. Some studies had reported some genes like ERG, PTEN, FOXA1 [2, 29, 30]. However, there is not enough study to point out why DM is associated with higher risk in Asia on genetic level, especially in East Asia, so we made a MR-analysis. We also considered the effects of life style, environment and food. Also, in the subgroup meta-analysis and meta-regression, the protective effect increases in the higher BMI population. This phenomenon aroused our interest. As is known to all, obesity is an important risk factor for cancer. But in prostate cancer, there are still some arguments about the influence of obesity. Some studies have reported the protective effect of obesity in prostate cancer [4, 31]. It was found that the obese man has a significantly lower risk of low-grade disease. A Mendelian randomization reported that testosterone mediates the protective effect of obesity [32]. Several researchers also think a higher BMI may lead to a lower concentration of PSA [33], and it is harder to detect prostate cancer in obese people [31, 34]. That may be a good explanation for the regional difference in results. In our study, the Asia population has a lower average BMI, and more prostate cancer can be detected in diabetes mellitus patients. And in the same population, a higher BMI is associated with a lower risk of prostate cancer in diabetes mellitus patients. However, more studies should be conducted to explain this phenomenon. Because obesity is associated with diabetes mellitus directly [35], and obese people may have some unhealthy lifestyles.

To continue exploring, we conducted a MR. Interestingly, we found a negative tendency of prostate cancer risk in European patients with diabetes mellitus after adjusted by BMI, though the result has no horizontal pleiotropy. And there was no statistical significance in the MR of the same population not adjusted by BMI. The MR makes the protective effect of diabetes mellitus more confirmed. However, in the Asia population, diabetes mellitus still showed a protective effect, which is different from meta-analysis. MR study using genetic variation to construct the instrumental variables of exposure, reducing real world confounders. The results suggest that racial differences in the effects of diabetes on prostate cancer incidence at genetic level may not be as large as previously thought. We also found that BMI adjustment had an impact on the results. The influence of BMI may explain this result and we observed a significant protective effect in higher BMI groups in two Asia studies [36, 37]. However, genomic alterations in Asian prostate cancer were poorly defined. [2] And there are not much open access GWAS data of Asian population, more high equality GWAS data need to be studied.

There are still some limitations in this study. First, the study lacks some detailed BMI data of the articles we included, so the results can be undetailed, and the conclusions are not exacted. Second, we ignored the differences between T1DM and T2DM because most studies didn’t distinguish them, which may cause some bias. Third, diabetes mellitus is associated with many factors, and they can also influence the risk of prostate cancer. Finally, the MR results are not so convincing in statistics, and more GWAS data should be used to further study.

This research finds the effects of weight on prostate cancer incidence risk in diabetes mellitus patients and discusses the region differences. More attention can be paid to the mechanism of these differences, and it may be helpful to the prevention or treatment of prostate cancer.

## CONCLUSIONS

Diabetes mellitus has a protective effect on prostate cancer, especially in the European population, and the obesity plays an important role in it. But more mechanisms behind this should be elucidated.

## Supplementary Material

Supplementary Figure 1

Supplementary Table 1

Supplementary Table 2

Supplementary Table 3

Supplementary Table 4

Supplementary Table 5

Supplementary Table 6

Supplementary Table 7

Supplementary Table 8

Supplementary Table 9
