# Supplementary material for: The association between diabetes mellitus and prostate cancer: a meta-analysis and Mendelian randomization
Source: Aging (Albany NY). 2024 Jun 4;16(11):9584–98. doi: 10.18632/aging.205886 (PMC11210264; doi:10.18632/aging.205886)
Supplement: Supplementary Figure 1 [file aging-16-205886-s001.pdf]

SUPPLEMENTARY FIGURE

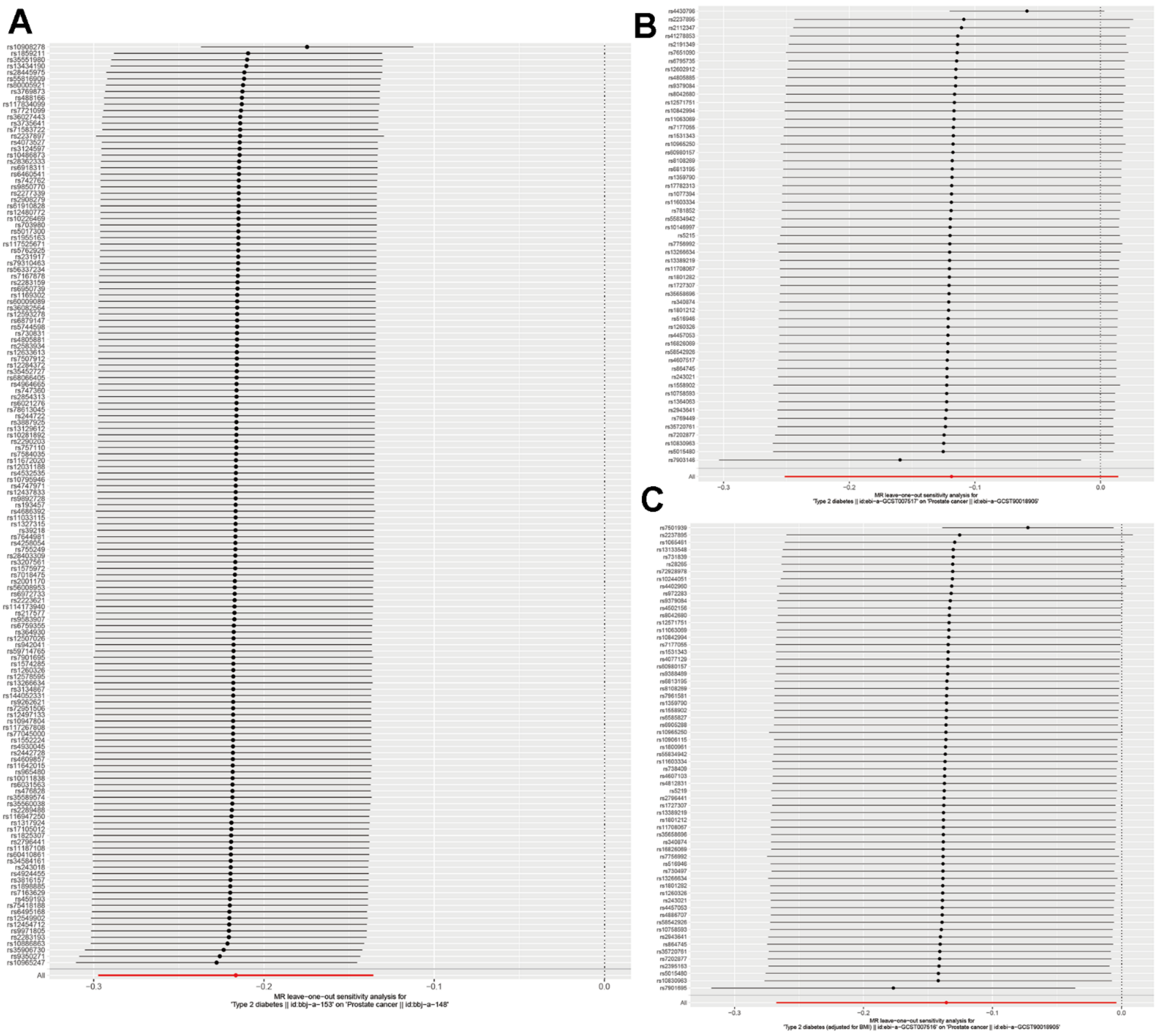

Supplementary Figure 1. The forest plot of Mendelian randomization. (**A**) The forest plot of MR in East Asia population. (**B**) The forest plot of MR in European population. (**C**) The forest plot of MR in European population adjusted by BMI.
