# Supplementary material for: The association between diabetes mellitus and prostate cancer: a meta-analysis and Mendelian randomization
Source: Aging (Albany NY). 2024 Jun 4;16(11):9584–98. doi: 10.18632/aging.205886 (PMC11210264; doi:10.18632/aging.205886)
Supplement: Supplementary Table 1 [file aging-16-205886-s002.pdf]

SUPPLEMENTARY TABLE

Supplementary Table 1A. Details of search strategy to retrieve the studies using PubMed (Medline).

Date of Search: 9/17/2022

#	Search terms	Hits
#1	Search: " Prostatic Neoplasms"[Mesh]	149563
#2	Search: (Prostate Neoplasms) OR (Neoplasms, Prostate) OR (Neoplasm, Prostate) OR (Prostate Neoplasm) OR (Neoplasms, Prostatic) OR (Neoplasm, Prostatic) OR (Prostatic Neoplasm) OR (Prostate Cancer) OR (Cancer, Prostate) OR (Cancers, Prostate) OR (Prostate Cancers) OR (Cancer of the Prostate) OR (Prostatic Cancer) OR (Cancer, Prostatic) OR (Cancers, Prostatic) OR (Prostatic Cancers) OR (Cancer of Prostate)	213661
#3	#1 OR #2	213661
#4	Search: " Diabetes Mellitus "[Mesh]	510469
#5	Search: (Diabetes) OR (Diabetes Mellitus)	948328
#6	#4 OR #5	948328
**#7**	**#3 and #6**	**3435**

Supplementary Table 1B. Details of search strategy to retrieve the studies using Embase.

Date of Search: 9/17/2022

#	Search terms	Hits
#1	'Diabetes' OR 'Diabetes Mellitus'	1579682
#2	'Prostate Neoplasms' OR 'Neoplasms, Prostate' OR 'Neoplasm, Prostate' OR 'Prostate Neoplasm' OR 'Neoplasms, Prostatic' OR 'Neoplasm, Prostatic' OR 'Prostatic Neoplasm' OR 'Prostate Cancer' OR 'Cancer, Prostate' OR 'Cancers, Prostate' OR 'Prostate Cancers' OR 'Cancer of the Prostate' OR 'Prostatic Cancer' OR 'Cancer, Prostatic' OR 'Cancers, Prostatic' OR 'Prostatic Cancers' OR 'Cancer of Prostate'	293405
**#3**	**#1 AND #2**	**8963**

Supplementary Table 1C. Details of search strategy to retrieve the studies using Cochrane.

Date of Search: 9/17/2022

#	Search terms	Hits
#1	((Diabetes) OR (Diabetes Mellitus)):ti,ab,kw	115706
#2	((Prostate Neoplasms) OR (Neoplasms, Prostate) OR (Neoplasm, Prostate) OR (Prostate Neoplasm) OR (Neoplasms, Prostatic) OR (Neoplasm, Prostatic) OR (Prostatic Neoplasm) OR (Prostate Cancer) OR (Cancer, Prostate) OR (Cancers, Prostate) OR (Prostate Cancers) OR (Cancer of the Prostate) OR (Prostatic Cancer) OR (Cancer, Prostatic) OR (Cancers, Prostatic) OR (Prostatic Cancers) OR (Cancer of Prostate)):ti,ab,kw	17564
**#3**	**#1 AND #2**	**2**
