# Supplementary material for: The association between diabetes mellitus and prostate cancer: a meta-analysis and Mendelian randomization
Source: Aging (Albany NY). 2024 Jun 4;16(11):9584–98. doi: 10.18632/aging.205886 (PMC11210264; doi:10.18632/aging.205886)
Supplement: Supplementary Table 2 [file aging-16-205886-s003.docx]

Supplementary Table 2. The GWAS ID and details of the included studies.

| **No.** | **id** | **trait** | **note** | **ncase** | **group_name** | **year** | **consortium** | **author** | **sex** | **population** | **unit** | **nsnp** | **sample_size** | **build** | **ncontrol** | **category** | **subcategory** | **ontology** | **mr** | **priority** | **pmid** | **sd** |
| --- | --- | --- | --- | --- | --- | --- | --- | --- | --- | --- | --- | --- | --- | --- | --- | --- | --- | --- | --- | --- | --- | --- |
| 1 | bbj-a-153 | Type 2 diabetes | NA | 40,250 | public | 2019 | NA | Ishigaki K | Males and Females | East Asian | NA | 8,885,694 | 210,865 | HG19/GRCh37 | 170,615 | Binary | NA | NA | 1 | 0 | NA | NA |
| 2 | bbj-a-148 | Prostate cancer | NA | 5,408 | public | 2019 | NA | Ishigaki K | Males and Females | East Asian | NA | 8,878,753 | 109,347 | HG19/GRCh37 | 103,939 | Binary | NA | NA | 1 | 0 | NA | NA |
| 3 | ebi-a-GCST007516 | Type 2 diabetes (adjusted for BMI) | NA | 48,286 | public | 2018 | NA | Mahajan A | NA | European | NA | 190,208 | 298,957 | HG19/GRCh37 | 250,671 | NA | NA | NA | 1 | 0 | 29632382 | NA |
| 4 | ebi-a-GCST007517 | Type 2 diabetes | NA | 48,286 | public | 2018 | NA | Mahajan A | NA | European | NA | 131,045 | 298,957 | HG19/GRCh37 | 250,671 | NA | NA | NA | 1 | 0 | 29632382 | NA |
| 5 | ebi-a-GCST90018905 | Prostate cancer | NA | 11,599 | public | 2,021 | NA | Sakaue S | NA | European | NA | 24,119,306 | 211,227 | HG19/GRCh37 | 199,628 | NA | NA | NA | 1 | 0 | 34594039 | NA |
