# Supplementary material for: The association between diabetes mellitus and prostate cancer: a meta-analysis and Mendelian randomization
Source: Aging (Albany NY). 2024 Jun 4;16(11):9584–98. doi: 10.18632/aging.205886 (PMC11210264; doi:10.18632/aging.205886)
Supplement: Supplementary Table 3 [file aging-16-205886-s004.docx]

Supplementary Table 3. Newcastle–Ottawa scale for assessing the quality of case-control studies in meta-analysis.

|  |  | **selection** |  |  |  | **Comparability** | **Exposure** |  |  |  |  |
| --- | --- | --- | --- | --- | --- | --- | --- | --- | --- | --- | --- |
| **Study** | **Year** | **1) Is the case definition adequate?** | **2) Representativeness of the cases** | **3) Selection of Controls** | **4) Definition of Controls** | **1) Comparability of cases and controls on the basis of the design or analysis** | **1) Ascertainment of exposure** | **2) Same method of ascertainment for cases and controls** | **3) Non-Response rate** | **Score** | **Note** |
| C. La Vecchia[1] | 1994 | 0 | 1 | 0 | 0 | 1 | 0 | 1 | 1 | 4 |  |
| Elizabeth A. Tindall[2] | 2013 | 0 | 1 | 0 | 0 | 1 | 0 | 1 | 1 | 4 |  |
| E. Lin[3] | 2020 | 1 | 1 | 0 | 1 | 1 | 1 | 1 | 0 | 6 |  |
| A. Tavani[4] | 2002 | 1 | 1 | 0 | 1 | 1 | 0 | 1 | 0 | 5 |  |
| Brandon L. Pierce[5] | 2008 | 1 | 1 | 1 | 0 | 2 | 0 | 1 | 0 | 6 |  |
| A. L. Coker[6] | 2004 | 1 | 0 | 1 | 0 | 2 | 0 | 1 | 0 | 5 |  |
| A. Tavani[7] | 2005 | 1 | 1 | 1 | 0 | 2 | 0 | 1 | 0 | 6 |  |
| Céline Lavalette[8] | 2022 | 0 | 1 | 0 | 1 | 0 | 1 | 1 | 0 | 4 |  |
| Clara Lam[9] | 2018 | 1 | 1 | 1 | 0 | 1 | 1 | 1 | 0 | 6 |  |
| Meng-Bo Hu[10] | 2019 | 1 | 0 | 0 | 0 | 2 | 1 | 1 | 0 | 5 |  |
| K. Zhu[11] | 2004 | 1 | 0 | 1 | 0 | 1 | 0 | 1 | 0 | 4 |  |
| Jesús Gibran Hernández-Pérez[12] | 2022 | 1 | 1 | 1 | 1 | 2 | 1 | 1 | 0 | 8 |  |
| Zhihong Gong[13] | 2006 | 1 | 0 | 0 | 0 | 1 | 1 | 1 | 0 | 4 |  |
| Gaurav Aggarwal[14] | 2013 | 1 | 1 | 0 | 0 | 0 | 0 | 1 | 1 | 4 |  |
| Rocío Barrios-Rodríguez[15] | 2022 | 1 | 1 | 0 | 0 | 2 | 0 | 1 | 1 | 6 |  |
| C. C. Hsieh[16] | 1999 | 0 | 1 | 0 | 1 | 1 | 0 | 1 | 1 | 5 |  |
| Saima Shakil Malik[17] | 2018 | 1 | 1 | 0 | 0 | 1 | 1 | 1 | 0 | 5 |  |
| B. Ganesh[18] | 2011 | 0 | 1 | 0 | 1 | 1 | 0 | 1 | 0 | 4 |  |
| Shih-Yi Lin[19] | 2021 | 1 | 1 | 1 | 0 | 2 | 1 | 1 | 0 | 7 |  |
| K. A. Moses[20] | 2012 | 0 | 1 | 0 | 0 | 2 | 1 | 1 | 0 | 5 |  |
| N. Baradaran[21] | 2009 | 0 | 1 | 0 | 1 | 1 | 0 | 1 | 1 | 5 |  |
| E. L. Turner[22] | 2011 | 1 | 1 | 0 | 0 | 1 | 0 | 1 | 0 | 4 |  |
| Rachael Williams[23] | 2018 | 1 | 1 | 1 | 0 | 2 | 1 | 1 | 0 | 7 |  |
| Cristina Bosetti[24] | 2012 | 0 | 1 | 0 | 1 | 2 | 0 | 1 | 1 | 6 |  |
| Marie-Claude Rousseau[25] | 2006 | 1 | 1 | 1 | 0 | 2 | 0 | 1 | 1 | 7 |  |
| Jennifer L. Beebe-Dimmer[26] | 2007 | 1 | 0 | 1 | 0 | 1 | 0 | 1 | 0 | 4 |  |
| Qiang Li[27] | 2010 | 1 | 1 | 0 | 1 | 2 | 1 | 1 | 0 | 7 |  |
| Sui-Foon Lo[28] | 2013 | 1 | 1 | 1 | 1 | 1 | 1 | 1 | 0 | 7 |  |
| P. Wallström [29] | 2009 | 1 | 1 | 0 | 1 | 2 | 1 | 1 | 1 | 8 |  |
| S.S Strom[30] | 2008 | 0 | 1 | 0 | 1 | 1 | 1 | 1 | 0 | 5 |  |
| Gholamreza Pourmand[31] | 2007 | 0 | 1 | 0 | 1 | 0 | 0 | 1 | 1 | 4 |  |
| Hsin-Chieh Yeh[32] | 2012 | 0 | 1 | 0 | 1 | 2 | 1 | 1 | 0 | 6 |  |
| A. González-Pérez[33] | 2005 | 1 | 1 | 0 | 0 | 2 | 1 | 1 | 0 | 6 |  |
| H.H. Bjornsdottir[34] | 2018 | 1 | 1 | 0 | 0 | 0 | 0 | 1 | 0 | 3 | Conference Abstract |
| C.B. Chen[35] | 2018 | 0 | 1 | 1 | 1 | 0 | 1 | 1 | 0 | 5 |  |

[1] C. La Vecchia, E. Negri, S. Franceschi, B. D’Avanzo, and P. Boyle, “A case-control study of diabetes mellitus and cancer risk,” *Br J Cancer*, vol. 70, no. 5, pp. 950–953, Nov. 1994, doi: 10.1038/bjc.1994.427.

[2] E. A. Tindall *et al.*, “Addressing the contribution of previously described genetic and epidemiological risk factors associated with increased prostate cancer risk and aggressive disease within men from South Africa,” *BMC Urol*, vol. 13, p. 74, Dec. 2013, doi: 10.1186/1471-2490-13-74.

[3] E. Lin *et al.*, “Association of type 2 diabetes mellitus and antidiabetic medication with risk of prostate cancer: a population-based case-control study,” *BMC Cancer*, vol. 20, no. 1, p. 551, Jun. 2020, doi: 10.1186/s12885-020-07036-4.

[4] A. Tavani *et al.*, “Diabetes and the risk of prostate cancer,” *Eur J Cancer Prev*, vol. 11, no. 2, pp. 125–128, Apr. 2002, doi: 10.1097/00008469-200204000-00003.

[5] B. L. Pierce, S. Plymate, E. A. Ostrander, and J. L. Stanford, “Diabetes mellitus and prostate cancer risk,” *Prostate*, vol. 68, no. 10, pp. 1126–1132, Jul. 2008, doi: 10.1002/pros.20777.

[6] A. L. Coker, M. Sanderson, W. Zheng, and M. K. Fadden, “Diabetes mellitus and prostate cancer risk among older men: population-based case-control study,” *Br J Cancer*, vol. 90, no. 11, pp. 2171–2175, Jun. 2004, doi: 10.1038/sj.bjc.6601857.

[7] A. Tavani *et al.*, “Diabetes mellitus and the risk of prostate cancer in Italy,” *Eur Urol*, vol. 47, no. 3, pp. 313–317; discussion 317, Mar. 2005, doi: 10.1016/j.eururo.2004.10.027.

[8] C. Lavalette *et al.*, “Diabetes, metabolic syndrome and prostate cancer risk: Results from the EPICAP case-control study,” *Cancer Epidemiol*, vol. 81, p. 102281, Oct. 2022, doi: 10.1016/j.canep.2022.102281.

[9] C. Lam, K. Cronin, R. Ballard, and A. Mariotto, “Differences in cancer survival among white and black cancer patients by presence of diabetes mellitus: Estimations based on SEER-Medicare-linked data resource,” *Cancer Med*, vol. 7, no. 7, pp. 3434–3444, Jul. 2018, doi: 10.1002/cam4.1554.

[10] M.-B. Hu *et al.*, “Effects of diabetes mellitus and Metformin administration on prostate cancer detection at biopsy among Chinese men: a case-control study,” *J BUON*, vol. 24, no. 1, pp. 227–232, 2019.

[11] K. Zhu, I.-M. Lee, H. D. Sesso, J. E. Buring, R. S. Levine, and J. M. Gaziano, “History of diabetes mellitus and risk of prostate cancer in physicians,” *Am J Epidemiol*, vol. 159, no. 10, pp. 978–982, May 2004, doi: 10.1093/aje/kwh139.

[12] J. G. Hernández-Pérez *et al.*, “Metabolic Syndrome and Prostate Cancer Risk: A Population Case-control Study,” *Arch Med Res*, vol. 53, no. 6, pp. 594–602, Sep. 2022, doi: 10.1016/j.arcmed.2022.07.003.

[13] Z. Gong *et al.*, “Obesity, diabetes, and risk of prostate cancer: results from the prostate cancer prevention trial,” *Cancer Epidemiol Biomarkers Prev*, vol. 15, no. 10, pp. 1977–1983, Oct. 2006, doi: 10.1158/1055-9965.EPI-06-0477.

[14] G. Aggarwal, P. Kamada, and S. T. Chari, “Prevalence of diabetes mellitus in pancreatic cancer compared to common cancers,” *Pancreas*, vol. 42, no. 2, pp. 198–201, Mar. 2013, doi: 10.1097/MPA.0b013e3182592c96.

[15] C. Rodriguez, A. V. Patel, A. M. Mondul, E. J. Jacobs, M. J. Thun, and E. E. Calle, “Diabetes and risk of prostate cancer in a prospective cohort of US men,” *Am J Epidemiol*, vol. 161, no. 2, pp. 147–152, Jan. 2005, doi: 10.1093/aje/kwh334.

[16] C. C. Hsieh, A. Thanos, D. Mitropoulos, C. Deliveliotis, C. S. Mantzoros, and D. Trichopoulos, “Risk factors for prostate cancer: a case-control study in Greece,” *Int J Cancer*, vol. 80, no. 5, pp. 699–703, Mar. 1999, doi: 10.1002/(sici)1097-0215(19990301)80:5<699::aid-ijc12>3.0.co;2-7.

[17] S. S. Malik, R. Batool, N. Masood, and A. Yasmin, “Risk factors for prostate cancer: A multifactorial case-control study,” *Curr Probl Cancer*, vol. 42, no. 3, pp. 337–343, 2018, doi: 10.1016/j.currproblcancer.2018.01.014.

[18] B. Ganesh, S. L. Saoba, M. N. Sarade, and S. V. Pinjari, “Risk factors for prostate cancer: An hospital-based case-control study from Mumbai, India,” *Indian J Urol*, vol. 27, no. 3, pp. 345–350, Jul. 2011, doi: 10.4103/0970-1591.85438.

[19] S.-Y. Lin *et al.*, “Risk of diabetes mellitus in patients with prostate cancer receiving injection therapy: A nationwide population-based propensity score-matched study,” *Int J Clin Pract*, vol. 75, no. 9, p. e14416, Sep. 2021, doi: 10.1111/ijcp.14416.

[20] K. A. Moses, O. A. Utuama, M. Goodman, and M. M. Issa, “The association of diabetes and positive prostate biopsy in a US veteran population,” *Prostate Cancer Prostatic Dis*, vol. 15, no. 1, pp. 70–74, Mar. 2012, doi: 10.1038/pcan.2011.40.

[21] N. Baradaran *et al.*, “The protective effect of diabetes mellitus against prostate cancer: role of sex hormones,” *Prostate*, vol. 69, no. 16, pp. 1744–1750, Dec. 2009, doi: 10.1002/pros.21023.

[22] E. L. Turner *et al.*, “Association of diabetes mellitus with prostate cancer: nested case-control study (Prostate testing for cancer and treatment study),” *Int J Cancer*, vol. 128, no. 2, pp. 440–446, Jan. 2011, doi: 10.1002/ijc.25360.

[23] R. Williams, T.-P. van Staa, A. M. Gallagher, T. Hammad, H. G. M. Leufkens, and F. de Vries, “Cancer recording in patients with and without type 2 diabetes in the Clinical Practice Research Datalink primary care data and linked hospital admission data: a cohort study,” *BMJ Open*, vol. 8, no. 5, p. e020827, May 2018, doi: 10.1136/bmjopen-2017-020827.

[24] C. Bosetti *et al.*, “Diabetes mellitus and cancer risk in a network of case-control studies,” *Nutr Cancer*, vol. 64, no. 5, pp. 643–651, 2012, doi: 10.1080/01635581.2012.676141.

[25] M.-C. Rousseau, M.-E. Parent, M. N. Pollak, and J. Siemiatycki, “Diabetes mellitus and cancer risk in a population-based case-control study among men from Montreal, Canada,” *Int J Cancer*, vol. 118, no. 8, pp. 2105–2109, Apr. 2006, doi: 10.1002/ijc.21600.

[26] J. L. Beebe-Dimmer, R. L. Dunn, A. V. Sarma, J. E. Montie, and K. A. Cooney, “Features of the metabolic syndrome and prostate cancer in African-American men,” *Cancer*, vol. 109, no. 5, pp. 875–881, Mar. 2007, doi: 10.1002/cncr.22461.

[27] Q. Li *et al.*, “History of diabetes mellitus and the risk of prostate cancer: the Ohsaki Cohort Study,” *Cancer Causes Control*, vol. 21, no. 7, pp. 1025–1032, Jul. 2010, doi: 10.1007/s10552-010-9530-9.

[28] S.-F. Lo *et al.*, “Modest increase in risk of specific types of cancer types in type 2 diabetes mellitus patients,” *Int J Cancer*, vol. 132, no. 1, pp. 182–188, Jan. 2013, doi: 10.1002/ijc.27597.

[29] P. Wallström, A. Bjartell, B. Gullberg, H. Olsson, and E. Wirfält, “A prospective Swedish study on body size, body composition, diabetes, and prostate cancer risk,” *Br J Cancer*, vol. 100, no. 11, pp. 1799–1805, Jun. 2009, doi: 10.1038/sj.bjc.6605077.

[30] S. S. Strom, Y. Yamamura, F. N. Flores-Sandoval, C. A. Pettaway, and D. S. Lopez, “Prostate cancer in Mexican-Americans: identification of risk factors,” *Prostate*, vol. 68, no. 5, pp. 563–570, Apr. 2008, doi: 10.1002/pros.20713.

[31] G. Pourmand *et al.*, “The risk factors of prostate cancer: a multicentric case-control study in Iran,” *Asian Pac J Cancer Prev*, vol. 8, no. 3, pp. 422–428, 2007.

[32] H.-C. Yeh, E. A. Platz, N.-Y. Wang, K. Visvanathan, K. J. Helzlsouer, and F. L. Brancati, “A prospective study of the associations between treated diabetes and cancer outcomes,” *Diabetes Care*, vol. 35, no. 1, pp. 113–118, Jan. 2012, doi: 10.2337/dc11-0255.

[33] A. González-Pérez and L. A. García Rodríguez, “Prostate cancer risk among men with diabetes mellitus (Spain),” *Cancer Causes Control*, vol. 16, no. 9, pp. 1055–1058, Nov. 2005, doi: 10.1007/s10552-005-4705-5.

[34] H. H. Bjornsdottir, S. Franzén, and A. Rawshani, “Cancer incidence and mortality among 457,473 persons with type 2 diabetes compared to 2,287,365 matched controls in Sweden: An observational study,” in *Diabetologia*, in English, vol. vol.61. 2018, p. S591. doi: 10.1007/s00125-018-4693-0.

[35] C. B. Chen, D. T. Eurich, S. R. Majumdar, and J. A. Johnson, “Risk of prostate cancer across different racial/ethnic groups in men with diabetes: a retrospective cohort study,” *Diabet Med*, vol. 35, no. 1, pp. 107–111, Jan. 2018, doi: 10.1111/dme.13536.
