# Supplementary material for: The association between diabetes mellitus and prostate cancer: a meta-analysis and Mendelian randomization
Source: Aging (Albany NY). 2024 Jun 4;16(11):9584–98. doi: 10.18632/aging.205886 (PMC11210264; doi:10.18632/aging.205886)
Supplement: Supplementary Table 4 [file aging-16-205886-s005.docx]

Supplementary Table 4. Newcastle–Ottawa scale for assessing the quality of cohort studies in meta-analysis.

|  |  | **selection** |  |  |  | **Comparability** | **Exposure** |  |  |  |  |
| --- | --- | --- | --- | --- | --- | --- | --- | --- | --- | --- | --- |
| **Study** | **Year** | **1) Representativeness of the exposed cohort** | **2) Selection of the non exposed cohort** | **3) Ascertainment of exposure** | **4) Demonstration that outcome of interest was not present at start of study** | **1) Comparability of cohorts on the basis of the design or analysis** | **1) Assessment of outcome** | **2) Was follow-up long enough for outcomes to occur** | **3) Adequacy of follow up of cohorts** | **Score** | **Note** |
| Y. R. Lawrence[1] | 2013 | 0 | 1 | 1 | 1 | 2 | 1 | 1 | 1 | 8 |  |
| Kevin M. Waters[2] | 2009 | 1 | 1 | 1 | 1 | 1 | 1 | 1 | 0 | 6 |  |
| Paola Ballotari[3] | 2017 | 1 | 1 | 1 | 1 | 1 | 1 | 0 | 1 | 6 |  |
| Gabriel Chodick[4] | 2010 | 1 | 0 | 1 | 1 | 2 | 1 | 1 | 1 | 7 |  |
| Carmen Rodriguez[5] | 2005 | 1 | 1 | 1 | 1 | 2 | 1 | 1 | 1 | 8 |  |
| C. M. Velicer[6] | 2007 | 1 | 1 | 1 | 1 | 2 | 1 | 0 | 1 | 7 |  |
| Michael F. Leitzmann[7] | 2008 | 1 | 1 | 1 | 1 | 2 | 1 | 1 | 1 | 8 |  |
| Konstantinos K. Tsilidis[8] | 2014 | 1 | 1 | 1 | 1 | 2 | 1 | 1 | 1 | 8 |  |
| Jocelyn S. Kasper[9] | 2009 | 0 | 1 | 1 | 1 | 2 | 0 | 1 | 1 | 7 |  |
| David J. Rosenberg[10] | 2002 | 1 | 1 | 1 | 0 | 1 | 1 | 1 | 1 | 6 |  |
| Aurora Perez-Cornago[11] | 2020 | 1 | 1 | 1 | 1 | 2 | 1 | 1 | 1 | 8 |  |
| Visalini Nair-Shalliker[12] | 2022 | 1 | 1 | 0 | 1 | 2 | 1 | 1 | 1 | 7 |  |
| Teemu J. Murtola[13] | 2018 | 1 | 1 | 0 | 1 | 2 | 1 | 1 | 0 | 6 |  |
| Jeanne A. Darbinian[14] | 2008 | 1 | 1 | 1 | 1 | 1 | 1 | 1 | 0 | 6 |  |
| Brook A. Calton[15] | 2007 | 1 | 1 | 1 | 1 | 2 | 0 | 1 | 1 | 7 |  |
| J. C. Will[16] | 1999 | 0 | 1 | 0 | 1 | 1 | 0 | 1 | 1 | 5 |  |
| Elizabeth A. Atchison[17] | 2011 | 0 | 1 | 1 | 0 | 2 | 1 | 1 | 1 | 7 |  |
| Birgitta Grundmark[18] | 2010 | 1 | 1 | 0 | 1 | 0 | 1 | 1 | 1 | 5 |  |
| Eric A. Miller[19] | 2018 | 1 | 1 | 1 | 1 | 2 | 1 | 1 | 1 | 8 |  |
| Hadith Rastad[20] | 2019 | 0 | 1 | 0 | 1 | 1 | 0 | 1 | 0 | 4 |  |
| Kozue Nakamura[21] | 2013 | 1 | 1 | 1 | 1 | 2 | 1 | 1 | 1 | 8 |  |
| Sheng-Hwu Hsieh[22] | 2014 | 0 | 1 | 1 | 0 | 0 | 1 | 0 | 1 | 4 |  |
| Bo Attner[23] | 2012 | 1 | 1 | 1 | 0 | 1 | 1 | 1 | 1 | 6 |  |
| H. O. Adami[24] | 1991 | 1 | 1 | 1 | 1 | 0 | 1 | 1 | 1 | 6 |  |
| Richard M. Martin[25] | 2009 | 1 | 1 | 1 | 1 | 2 | 1 | 1 | 1 | 8 |  |
| C. Wu[26] | 2011 | 1 | 0 | 1 | 0 | 2 | 1 | 0 | 1 | 5 |  |
| Dianna J. Magliano[27] | 2012 | 1 | 0 | 1 | 0 | 1 | 1 | 1 | 0 | 4 |  |
| Lauren P. Wallner[28] | 2011 | 1 | 1 | 0 | 1 | 0 | 1 | 1 | 1 | 5 |  |
| Bendix Carstensen[29] | 2016 | 1 | 0 | 1 | 1 | 0 | 1 | 1 | 0 | 4 |  |
| Mingyang Song[30] | 2020 | 0 | 1 | 1 | 1 | 2 | 0 | 1 | 0 | 6 |  |
| J.J. Walker [31] | 2013 | 0 | 0 | 1 | 1 | 1 | 1 | 1 | 0 | 5 |  |

[1] Y. R. Lawrence *et al.*, “Association between metabolic syndrome, diabetes mellitus and prostate cancer risk,” *Prostate Cancer Prostatic Dis*, vol. 16, no. 2, pp. 181–186, Jun. 2013, doi: 10.1038/pcan.2012.54.

[2] K. M. Waters, B. E. Henderson, D. O. Stram, P. Wan, L. N. Kolonel, and C. A. Haiman, “Association of diabetes with prostate cancer risk in the multiethnic cohort,” *Am J Epidemiol*, vol. 169, no. 8, pp. 937–945, Apr. 2009, doi: 10.1093/aje/kwp003.

[3] P. Ballotari *et al.*, “Diabetes and risk of cancer incidence: results from a population-based cohort study in northern Italy,” *BMC Cancer*, vol. 17, no. 1, p. 703, Oct. 2017, doi: 10.1186/s12885-017-3696-4.

[4] G. Chodick *et al.*, “Diabetes and risk of incident cancer: a large population-based cohort study in Israel,” *Cancer Causes Control*, vol. 21, no. 6, pp. 879–887, Jun. 2010, doi: 10.1007/s10552-010-9515-8.

[5] C. Rodriguez, A. V. Patel, A. M. Mondul, E. J. Jacobs, M. J. Thun, and E. E. Calle, “Diabetes and risk of prostate cancer in a prospective cohort of US men,” *Am J Epidemiol*, vol. 161, no. 2, pp. 147–152, Jan. 2005, doi: 10.1093/aje/kwh334.

[6] C. M. Velicer, S. Dublin, and E. White, “Diabetes and the risk of prostate cancer: the role of diabetes treatment and complications,” *Prostate Cancer Prostatic Dis*, vol. 10, no. 1, pp. 46–51, 2007, doi: 10.1038/sj.pcan.4500914.

[7] M. F. Leitzmann *et al.*, “Diabetes mellitus and prostate cancer risk in the Prostate, Lung, Colorectal, and Ovarian Cancer Screening Trial,” *Cancer Causes Control*, vol. 19, no. 10, pp. 1267–1276, Dec. 2008, doi: 10.1007/s10552-008-9198-6.

[8] K. K. Tsilidis *et al.*, “Diabetes mellitus and risk of prostate cancer in the European Prospective Investigation into Cancer and Nutrition,” *Int J Cancer*, vol. 136, no. 2, pp. 372–381, Jan. 2015, doi: 10.1002/ijc.28989.

[9] J. S. Kasper, Y. Liu, and E. Giovannucci, “Diabetes mellitus and risk of prostate cancer in the health professionals follow-up study,” *Int J Cancer*, vol. 124, no. 6, pp. 1398–1403, Mar. 2009, doi: 10.1002/ijc.24044.

[10] D. J. Rosenberg, A. I. Neugut, H. Ahsan, and S. Shea, “Diabetes mellitus and the risk of prostate cancer,” *Cancer Invest*, vol. 20, no. 2, pp. 157–165, 2002, doi: 10.1081/cnv-120001141.

[11] A. Perez-Cornago *et al.*, “Examination of potential novel biochemical factors in relation to prostate cancer incidence and mortality in UK Biobank,” *Br J Cancer*, vol. 123, no. 12, pp. 1808–1817, Dec. 2020, doi: 10.1038/s41416-020-01081-3.

[12] V. Nair-Shalliker *et al.*, “Family history, obesity, urological factors and diabetic medications and their associations with risk of prostate cancer diagnosis in a large prospective study,” *Br J Cancer*, vol. 127, no. 4, pp. 735–746, Sep. 2022, doi: 10.1038/s41416-022-01827-1.

[13] T. J. Murtola *et al.*, “Fasting blood glucose, glycaemic control and prostate cancer risk in the Finnish Randomized Study of Screening for Prostate Cancer,” *Br J Cancer*, vol. 118, no. 9, pp. 1248–1254, May 2018, doi: 10.1038/s41416-018-0055-4.

[14] J. A. Darbinian, A. M. Ferrara, S. K. Van Den Eeden, C. P. Quesenberry, B. Fireman, and L. A. Habel, “Glycemic status and risk of prostate cancer,” *Cancer Epidemiol Biomarkers Prev*, vol. 17, no. 3, pp. 628–635, Mar. 2008, doi: 10.1158/1055-9965.EPI-07-2610.

[15] B. A. Calton *et al.*, “History of diabetes mellitus and subsequent prostate cancer risk in the NIH-AARP Diet and Health Study,” *Cancer Causes Control*, vol. 18, no. 5, pp. 493–503, Jun. 2007, doi: 10.1007/s10552-007-0126-y.

[16] J. C. Will, F. Vinicor, and E. E. Calle, “Is diabetes mellitus associated with prostate cancer incidence and survival?,” *Epidemiology*, vol. 10, no. 3, pp. 313–318, May 1999.

[17] E. A. Atchison, G. Gridley, J. D. Carreon, M. F. Leitzmann, and K. A. McGlynn, “Risk of cancer in a large cohort of U.S. veterans with diabetes,” *Int J Cancer*, vol. 128, no. 3, pp. 635–643, Feb. 2011, doi: 10.1002/ijc.25362.

[18] B. Grundmark, H. Garmo, M. Loda, C. Busch, L. Holmberg, and B. Zethelius, “The metabolic syndrome and the risk of prostate cancer under competing risks of death from other causes,” *Cancer Epidemiol Biomarkers Prev*, vol. 19, no. 8, pp. 2088–2096, Aug. 2010, doi: 10.1158/1055-9965.EPI-10-0112.

[19] E. A. Miller, P. F. Pinsky, and D. Pierre-Victor, “The relationship between diabetes, prostate-specific antigen screening tests, and prostate cancer,” *Cancer Causes Control*, vol. 29, no. 10, pp. 907–914, Oct. 2018, doi: 10.1007/s10552-018-1067-3.

[20] H. Rastad, M. Parsaeian, N. Shirzad, M. A. Mansournia, and K. Yazdani, “Diabetes mellitus and cancer incidence: the Atherosclerosis Risk in Communities (ARIC) cohort study,” *J Diabetes Metab Disord*, vol. 18, no. 1, pp. 65–72, Jun. 2019, doi: 10.1007/s40200-019-00391-5.

[21] K. Nakamura *et al.*, “Diabetes mellitus and risk of cancer in Takayama: a population-based prospective cohort study in Japan,” *Cancer Sci*, vol. 104, no. 10, pp. 1362–1367, Oct. 2013, doi: 10.1111/cas.12235.

[22] S.-H. Hsieh, W.-K. Chiou, M.-H. Wang, and J.-D. Lin, “Association of body weight with the risk for malignancies in hospitalized patients with or without diabetes mellitus in Taiwan,” *J Investig Med*, vol. 62, no. 1, pp. 37–42, Jan. 2014, doi: 10.2310/JIM.0000000000000004.

[23] B. Attner, M. Landin-Olsson, T. Lithman, D. Noreen, and H. Olsson, “Cancer among patients with diabetes, obesity and abnormal blood lipids: a population-based register study in Sweden,” *Cancer Causes Control*, vol. 23, no. 5, pp. 769–777, May 2012, doi: 10.1007/s10552-012-9946-5.

[24] H. O. Adami *et al.*, “Cancer risk in patients with diabetes mellitus,” *Cancer Causes Control*, vol. 2, no. 5, pp. 307–314, Sep. 1991, doi: 10.1007/BF00051670.

[25] R. M. Martin, L. Vatten, D. Gunnell, P. Romundstad, and T. I. L. Nilsen, “Components of the metabolic syndrome and risk of prostate cancer: the HUNT 2 cohort, Norway,” *Cancer Causes Control*, vol. 20, no. 7, pp. 1181–1192, Sep. 2009, doi: 10.1007/s10552-009-9319-x.

[26] C. Wu, D. M. Moreira, L. Gerber, R. S. Rittmaster, G. L. Andriole, and S. J. Freedland, “Diabetes and prostate cancer risk in the REDUCE trial,” *Prostate Cancer Prostatic Dis*, vol. 14, no. 4, pp. 326–331, Dec. 2011, doi: 10.1038/pcan.2011.28.

[27] D. J. Magliano, W. A. Davis, J. E. Shaw, D. G. Bruce, and T. M. E. Davis, “Incidence and predictors of all-cause and site-specific cancer in type 2 diabetes: the Fremantle Diabetes Study,” *Eur J Endocrinol*, vol. 167, no. 4, pp. 589–599, Oct. 2012, doi: 10.1530/EJE-12-0053.

[28] L. P. Wallner *et al.*, “The effects of metabolic conditions on prostate cancer incidence over 15 years of follow-up: results from the Olmsted County Study,” *BJU Int*, vol. 107, no. 6, pp. 929–935, Mar. 2011, doi: 10.1111/j.1464-410X.2010.09703.x.

[29] B. Carstensen *et al.*, “Cancer incidence in persons with type 1 diabetes: a five-country study of 9,000 cancers in type 1 diabetic individuals,” *Diabetologia*, vol. 59, no. 5, pp. 980–988, May 2016, doi: 10.1007/s00125-016-3884-9.

[30] Y. Hu *et al.*, “Incident Type 2 Diabetes Duration and Cancer Risk: A Prospective Study in Two US Cohorts,” *J Natl Cancer Inst*, vol. 113, no. 4, pp. 381–389, Apr. 2021, doi: 10.1093/jnci/djaa141.

[31] J. J. Walker *et al.*, “Type 2 diabetes, socioeconomic status and risk of cancer in Scotland 2001-2007,” *Diabetologia*, vol. 56, no. 8, pp. 1712–1715, Aug. 2013, doi: 10.1007/s00125-013-2937-6.
