# Supplementary material for: The association between diabetes mellitus and prostate cancer: a meta-analysis and Mendelian randomization
Source: Aging (Albany NY). 2024 Jun 4;16(11):9584–98. doi: 10.18632/aging.205886 (PMC11210264; doi:10.18632/aging.205886)
Supplement: Supplementary Table 5 [file aging-16-205886-s006.docx]

Supplementary Table 5. Agency for healthcare research and quality (AHRQ) criteria for assessing the quality of cross‐sectional studies in meta-analysis.

| **Study** | **Year** | **Define the source of information (survey, record review)** | **List inclusion and exclusion criteria for exposed and unexposed subjects (cases and controls) or refer to previous publications** | **Indicate time period used for identifying patients** | **Indicate whether or not subjects were consectutive if not population-based** | **Indicate if evaluators of subjective components of study were masked to other aspects of the status of the participants** | **Describle any assessments undertaken for quality assurance purposes (e.g., test/retest of primary outcome measurements)** | **Explain any patient exclusions from analysis** | **Describle how confounding was assessed and/or controlled** | **If applicable, explain how missing data were handled in the analysis** | **Summarize patient response rates and completeness of data collection** | **Clarify what follow-up, if any, was expected and the percentage of patients for which incomplete data of follow-up was obtained** | **Score** |
| --- | --- | --- | --- | --- | --- | --- | --- | --- | --- | --- | --- | --- | --- |
| Maria Camila Suarez Arbelaez[1] | 2023 | 1 | 1 | 1 | 1 | 0 | 1 | 0 | 1 | 0 | 0 | 0 | 6 |
| Chin-Hsiao Tseng[2] | 2011 | 1 | 1 | 1 | 1 | 0 | 1 | 1 | 0 | 0 | 1 | 0 | 7 |
| Chaoyang Li[3] | 2011 | 1 | 1 | 1 | 1 | 0 | 0 | 1 | 1 | 0 | 0 | 0 | 6 |
| Cuiping Bao[4] | 2018 | 1 | 1 | 1 | 1 | 0 | 1 | 1 | 1 | 1 | 1 | 0 | 9 |
| Lourdes Guerrios-Rivera[5] | 2023 | 1 | 1 | 1 | 0 | 0 | 0 | 1 | 1 | 0 | 1 | 0 | 6 |
| N. J. Karlin[6] | 2023 | 1 | 1 | 1 | 1 | 0 | 1 | 1 | 1 | 1 | 0 | 0 | 8 |

[1] M. C. Suarez Arbelaez *et al.*, “Association between body mass index, metabolic syndrome and common urologic conditions: a cross-sectional study using a large multi-institutional database from the United States,” *Ann Med*, vol. 55, no. 1, p. 2197293, Dec. 2023, doi: 10.1080/07853890.2023.2197293.

[2] C.-H. Tseng, “Diabetes and risk of prostate cancer: a study using the National Health Insurance,” *Diabetes Care*, vol. 34, no. 3, pp. 616–621, Mar. 2011, doi: 10.2337/dc10-1640.

[3] C. Li, L. S. Balluz, E. S. Ford, C. A. Okoro, J. Tsai, and G. Zhao, “Association between diagnosed diabetes and self-reported cancer among U.S. adults: findings from the 2009 Behavioral Risk Factor Surveillance System,” *Diabetes Care*, vol. 34, no. 6, pp. 1365–1368, Jun. 2011, doi: 10.2337/dc11-0020.

[4] C. Bao *et al.*, “Diabetes in midlife and risk of cancer in late life: A nationwide Swedish twin study,” *Int J Cancer*, vol. 143, no. 4, pp. 793–800, Aug. 2018, doi: 10.1002/ijc.31365.

[5] L. Guerrios-Rivera *et al.*, “Metabolic syndrome is associated with aggressive prostate cancer regardless of race,” *Cancer Causes Control*, vol. 34, no. 3, pp. 213–221, Mar. 2023, doi: 10.1007/s10552-022-01649-9.

[6] N. J. Karlin, H. E. Kosiorek, P. M. Verona, K. E. Coppola, and C. B. Cook, “Cancer, diabetes, survival and glycemic control: a large multisite analysis,” *Future Sci OA*, vol. 8, no. 9, p. FSO820, Oct. 2022, doi: 10.2144/fsoa-2022-0018.
