# Supplementary material for: The association between diabetes mellitus and prostate cancer: a meta-analysis and Mendelian randomization
Source: Aging (Albany NY). 2024 Jun 4;16(11):9584–98. doi: 10.18632/aging.205886 (PMC11210264; doi:10.18632/aging.205886)
Supplement: Supplementary Table 6 [file aging-16-205886-s007.docx]

Supplementary Table 6. The outcome, heterogeneity and pleiotropy of MR (id: bbj-a-153, bbj-a-148).

| Outcome |  |  |  |  |  |  |  |  |  |
| --- | --- | --- | --- | --- | --- | --- | --- | --- | --- |
|  | id.exposure | id.outcome | outcome | exposure | method | nsnp | b | se | pval |
| 1 | bbj-a-153 | bbj-a-148 | Prostate cancer \|\| id:bbj-a-148 | Type 2 diabetes \|\| id:bbj-a-153 | MR Egger | 145 | -0.24964 | 0.10177 | 0.01537 |
| 2 | bbj-a-153 | bbj-a-148 | Prostate cancer \|\| id:bbj-a-148 | Type 2 diabetes \|\| id:bbj-a-153 | Weighted median | 145 | -0.16355 | 0.04203 | 1E-04 |
| 3 | bbj-a-153 | bbj-a-148 | Prostate cancer \|\| id:bbj-a-148 | Type 2 diabetes \|\| id:bbj-a-153 | Inverse variance weighted | 145 | -0.21652 | 0.04123 | 1.5E-07 |
| 4 | bbj-a-153 | bbj-a-148 | Prostate cancer \|\| id:bbj-a-148 | Type 2 diabetes \|\| id:bbj-a-153 | Simple mode | 145 | -0.25089 | 0.09956 | 0.01283 |
| 5 | bbj-a-153 | bbj-a-148 | Prostate cancer \|\| id:bbj-a-148 | Type 2 diabetes \|\| id:bbj-a-153 | Weighted mode | 145 | -0.17178 | 0.06273 | 0.00696 |

| heterogeneity | |  |  |  |  |  |  |  |
| --- | --- | --- | --- | --- | --- | --- | --- | --- |
|  | id.exposure | id.outcome | outcome | exposure | method | Q | Q_df | Q_pval |
| 1 | bbj-a-153 | bbj-a-148 | Prostate cancer \|\| id:bbj-a-148 | Type 2 diabetes \|\| id:bbj-a-153 | MR Egger | 384.298 | 143 | 5.5E-24 |
| 2 | bbj-a-153 | bbj-a-148 | Prostate cancer \|\| id:bbj-a-148 | Type 2 diabetes \|\| id:bbj-a-153 | Inverse variance weighted | 384.639 | 144 | 8.1E-24 |

| pleiotropy | |  |  |  |  |  |  |
| --- | --- | --- | --- | --- | --- | --- | --- |
|  | id.exposure | id.outcome | outcome | exposure | egger_intercept | se | pval |
| 1 | bbj-a-153 | bbj-a-148 | Prostate cancer \|\| id:bbj-a-148 | Type 2 diabetes \|\| id:bbj-a-153 | 0.00295 | 0.00827 | 0.72221 |
