# Supplementary material for: The association between diabetes mellitus and prostate cancer: a meta-analysis and Mendelian randomization
Source: Aging (Albany NY). 2024 Jun 4;16(11):9584–98. doi: 10.18632/aging.205886 (PMC11210264; doi:10.18632/aging.205886)
Supplement: Supplementary Table 7 [file aging-16-205886-s008.docx]

Supplementary Table 7. The outcome, heterogeneity and pleiotropy of MR (id: ebi-a-GCST007517, ebi-a-GCST90018905).

| Outcome |  |  |  |  |  |  |  |  |  |
| --- | --- | --- | --- | --- | --- | --- | --- | --- | --- |
|  | id.exposure | id.outcome | outcome | exposure | method | nsnp | b | se | pval |
| 1 | ebi-a-GCST007517 | ebi-a-GCST90018905 | Prostate cancer \|\| id:ebi-a-GCST90018905 | Type 2 diabetes \|\| id:ebi-a-GCST007517 | MR Egger | 55 | 0.08016 | 0.16906 | 0.63735 |
| 2 | ebi-a-GCST007517 | ebi-a-GCST90018905 | Prostate cancer \|\| id:ebi-a-GCST90018905 | Type 2 diabetes \|\| id:ebi-a-GCST007517 | Weighted median | 55 | -0.05696 | 0.04063 | 0.1609 |
| 3 | ebi-a-GCST007517 | ebi-a-GCST90018905 | Prostate cancer \|\| id:ebi-a-GCST90018905 | Type 2 diabetes \|\| id:ebi-a-GCST007517 | Inverse variance weighted | 55 | -0.11865 | 0.06769 | 0.07964 |
| 4 | ebi-a-GCST007517 | ebi-a-GCST90018905 | Prostate cancer \|\| id:ebi-a-GCST90018905 | Type 2 diabetes \|\| id:ebi-a-GCST007517 | Simple mode | 55 | 0.01309 | 0.0751 | 0.86226 |
| 5 | ebi-a-GCST007517 | ebi-a-GCST90018905 | Prostate cancer \|\| id:ebi-a-GCST90018905 | Type 2 diabetes \|\| id:ebi-a-GCST007517 | Weighted mode | 55 | 0.03973 | 0.05243 | 0.45189 |

| heterogeneity | |  |  |  |  |  |  |  |
| --- | --- | --- | --- | --- | --- | --- | --- | --- |
|  | id.exposure | id.outcome | outcome | exposure | method | Q | Q_df | Q_pval |
| 1 | ebi-a-GCST007517 | ebi-a-GCST90018905 | Prostate cancer \|\| id:ebi-a-GCST90018905 | Type 2 diabetes \|\| id:ebi-a-GCST007517 | MR Egger | 345.634 | 53 | 1.5E-44 |
| 2 | ebi-a-GCST007517 | ebi-a-GCST90018905 | Prostate cancer \|\| id:ebi-a-GCST90018905 | Type 2 diabetes \|\| id:ebi-a-GCST007517 | Inverse variance weighted | 356.349 | 54 | 4E-46 |

| pleiotropy | |  |  |  |  |  |  |
| --- | --- | --- | --- | --- | --- | --- | --- |
|  | id.exposure | id.outcome | outcome | exposure | egger_intercept | se | pval |
| 1 | ebi-a-GCST007517 | ebi-a-GCST90018905 | Prostate cancer \|\| id:ebi-a-GCST90018905 | Type 2 diabetes \|\| id:ebi-a-GCST007517 | -0.01557 | 0.01215 | 0.20548 |
