# Supplementary material for: The association between diabetes mellitus and prostate cancer: a meta-analysis and Mendelian randomization
Source: Aging (Albany NY). 2024 Jun 4;16(11):9584–98. doi: 10.18632/aging.205886 (PMC11210264; doi:10.18632/aging.205886)
Supplement: Supplementary Table 8 [file aging-16-205886-s009.docx]

Supplementary Table 8. The outcome, heterogeneity and pleiotropy of MR (id: ebi-a-GCST007516, ebi-a-GCST90018905).

| Outcome |  |  |  |  |  |  |  |  |  |
| --- | --- | --- | --- | --- | --- | --- | --- | --- | --- |
|  | id.exposure | id.outcome | outcome | exposure | method | nsnp | b | se | pval |
| 1 | ebi-a-GCST007516 | ebi-a-GCST90018905 | Prostate cancer \|\| id:ebi-a-GCST90018905 | Type 2 diabetes (adjusted for BMI) \|\| id:ebi-a-GCST007516 | MR Egger | 64 | 0.04649 | 0.16018 | 0.77258 |
| 2 | ebi-a-GCST007516 | ebi-a-GCST90018905 | Prostate cancer \|\| id:ebi-a-GCST90018905 | Type 2 diabetes (adjusted for BMI) \|\| id:ebi-a-GCST007516 | Weighted median | 64 | -0.05115 | 0.04462 | 0.25162 |
| 3 | ebi-a-GCST007516 | ebi-a-GCST90018905 | Prostate cancer \|\| id:ebi-a-GCST90018905 | Type 2 diabetes (adjusted for BMI) \|\| id:ebi-a-GCST007516 | Inverse variance weighted | 64 | -0.13575 | 0.06722 | 0.04342 |
| 4 | ebi-a-GCST007516 | ebi-a-GCST90018905 | Prostate cancer \|\| id:ebi-a-GCST90018905 | Type 2 diabetes (adjusted for BMI) \|\| id:ebi-a-GCST007516 | Simple mode | 64 | 0.00396 | 0.07457 | 0.95786 |
| 5 | ebi-a-GCST007516 | ebi-a-GCST90018905 | Prostate cancer \|\| id:ebi-a-GCST90018905 | Type 2 diabetes (adjusted for BMI) \|\| id:ebi-a-GCST007516 | Weighted mode | 64 | 0.02292 | 0.05889 | 0.69843 |

| heterogeneity | |  |  |  |  |  |  |  |
| --- | --- | --- | --- | --- | --- | --- | --- | --- |
|  | id.exposure | id.outcome | outcome | exposure | method | Q | Q_df | Q_pval |
| 1 | ebi-a-GCST007516 | ebi-a-GCST90018905 | Prostate cancer \|\| id:ebi-a-GCST90018905 | Type 2 diabetes (adjusted for BMI) \|\| id:ebi-a-GCST007516 | MR Egger | 382.915 | 62 | 9.2E-48 |
| 2 | ebi-a-GCST007516 | ebi-a-GCST90018905 | Prostate cancer \|\| id:ebi-a-GCST90018905 | Type 2 diabetes (adjusted for BMI) \|\| id:ebi-a-GCST007516 | Inverse variance weighted | 392.6 | 63 | 3.9E-49 |

| pleiotropy | |  |  |  |  |  |  |
| --- | --- | --- | --- | --- | --- | --- | --- |
|  | id.exposure | id.outcome | outcome | exposure | egger_intercept | se | pval |
| 1 | ebi-a-GCST007516 | ebi-a-GCST90018905 | Prostate cancer \|\| id:ebi-a-GCST90018905 | Type 2 diabetes (adjusted for BMI) \|\| id:ebi-a-GCST007516 | -0.01301 | 0.01039 | 0.21517 |
