# Supplementary material for: The association between diabetes mellitus and prostate cancer: a meta-analysis and Mendelian randomization
Source: Aging (Albany NY). 2024 Jun 4;16(11):9584–98. doi: 10.18632/aging.205886 (PMC11210264; doi:10.18632/aging.205886)
Supplement: Supplementary Table 9 [file aging-16-205886-s010.docx]

Supplementary Table 9. Characteristics of included studies in this systematic review and meta‐analysis.

| **Study** | **Year** | **Region** | **Study-type** | **Patient characteristics** | **Definition of prostate cancer** | | **Age (years), median (range) or mean (SD)** | | | **Covariate adjustment** |
| --- | --- | --- | --- | --- | --- | --- | --- | --- | --- | --- |
| C. La Vecchia et al.^38^ | 1994 | Italy | case-control | An integrated series of case-control studies conducted in Northern Italy | Histologically confirmed neoplasms | | Under 75 | | | Adjusted for sex (when appropriate), age, education, smoking and body mass index |
| Elizabeth A. Tindall et al.^39^ | 2013 | South Africa | case-control | The Southern African Prostate Cancer Study (SAPCS) | Reviewed by local urologists, PSA testing performed | | Case= 71(49-101) Control= 70(45-99) | | | Adjusted for sex age, population and a family history of prostate cancer |
| Maria Camila Suarez Arbelaez et al.^40^ | 2023 | US | cross-section | TriNetX Research Network. | Urological conditions was determined in TriNetX. | | Case= 65 ±13 Control= 40 ±23 | | | NR |
| Y. R. Lawrence et al.^41^ | 2013 | Israeli | cohort | 12529 men and 2995 woman aged 36 to 74 years with known coronary heart disease were screened for the trial in 18 Israeli medical centers. | From the Israeli National Cancer Registry (INCR) | | Case= 59.3 ±7.3 Control= 60.5 ±6.5 | | | Adjusted for BMI, body mass index; DM, diabetes mellitus type II; HDL, high-density lipoprotein; MS, metabolic syndrome |
| Kevin M. Waters et al.^42^ | 2009 | US | cohort | The Multiethnic Cohort conducted in Hawaii and Los Angeles, California (African Americans, European Americans, Native Hawaiians, Japanese Americans, and Latinos). | Identified annually through cohort linkage to the population-based Surveillance, Epidemiology, and End Results (SEER) cancer registries in Hawaii and Los Angeles County, as well as the California Cancer Registry | | Exposure= 61.7±7.0 control =59.7±7.8 | | | Adjusted for age, body mass index, and educational level. Adjusted for race in pooled analysis. |
| E. Lin et al.^43^ | 2020 | Sweden | case-control | PCBaSe 4.1 based on the National Prostate Cancer Register (NPCR) of Sweden | All participants have been subsequently linked to Swedish health care registers and demographic databases. | | Reported | | | Adjusted for CCI, education level, civil status and the age at year of PCa onset. |
| Paola Ballotari et al.^44^ | 2017 | Italy | cohort | Reggio Emilia diabetes registry | Using the first incident cancer of each site during follow up and identifying it according to Reggio Emilia cancer registry. | | Exposure= 67(58-75) Control = 45(35-60) | | | Adjusted for age, foreign status, and sex, using Poisson model. |
| Gabriel Chodick et al.^45^ | 2010 | Israeli | cohort | The MHS registry of DM patients. | Data on cancer occurrence during study follow-up period were obtained from the Israel Cancer Register (ICR) | | Exposure= 61.6±13.1 Control= 61.6±13.4 | | | Adjusted for age, region, SES level, use of healthcare services a year prior to index date, BMI, and history of cardiovascular diseases. |
| Carmen Rodriguez et al.^46^ | 2005 | US | cohort | Participants in the study completed a mailed questionnaire including information on diabetes at enrollment in 1992 and at follow-up questionnaires in 1997 and 1999. | Identified through a self-report of cancer on any of the questionnaires and subsequently verified by medical records or from linkage with state cancer registries | | Reported | | | Adjusted for age at entry, race, education, body mass index, family history of prostate cancer, prostate-specific antigen, quintiles of total fat, and quintiles of energy-adjusted intakes of the following: lycopene, total calcium, and total fat. |
| Chin-Hsiao Tseng^12^ | 2011 | China(TW) | cross-section | A random sample of 1,000,000 subjects covered by the National Health Insurance in 2005. | NR | | More than 40 | | | NR |
| A. Tavani et al.^47^ | 2002 | Italy and Greece | case-control | Data were derived from two hospital-based case–control studies on prostate cancer, conducted in Italy and Greece | Histologically confirmed | | Italy Case= 67 Control= 60 Greece Case= 71 Control= 70 | | | Adjusted for centre, calendar ear at interview,age,education and body mass index. |
| C. M. Velicer et al.^48^ | 2007 | US | cohort | A prospective cohort study of 35 239 men, 50–76 years old, in Washington State who completed a baseline questionnaire between 2000 and 2002. | Identified through the SEER registry | | Exposure= 64.3 Control= 61.5 | | | Adjusted for age and PSA testing. |
| Brandon L. Pierce et al.^49^ | 2008 | US | case-control | Cases and controls were residents of King County, Washington identified using the Surveillance, Epidemiology, and End Results Seattle-Puget Sound cancer registry and random digit dialing | Identified through the SEER registry | | Under 74 | | | Adjusted for categorical age, PCa screening history, categorical BMI, race, study, and family hist |
| A. L. Coker et al.^50^ | 2004 | US | case-control | A population-based case–control study of 407 incident histologically confirmed cases registered in the South Carolina Central Cancer Registry between 1999 and 2001; controls were 393 men identified through the Health Care Financing Administration Medicare beneficiary file for South Carolina in 1999 | Diagnosed with primary invasive histologically confirmed prostate cancer | | 65-79 | | | Adjusted for age (categorical variable), race (African-American compared with Whites), South Carolina region (three areas), and prostate cancer screening in the past 5 years before the referent date (yes annual screening vs no). |
| Michael F. Leitzmann et al.^51^ | 2008 | US | cohort | Prostate, Lung, Colorectal, and Ovarian (PLCO) Cancer Screening Trial | Medical records to confirm the diagnosis and to obtain stage and grade information. | | Exposure= 64.0(59.0-68.0) Control =62.0(58.0-66.0) | | | Adjusted for age, race, family history of prostate cancer, study center, aspirin use, education, smoking, body mass index, physical activity, and height. Number of cases by grade or stage may not sum to total cases due to missing information on grade or stage |
| Konstantinos K. Tsilidis et al.^52^ | 2014 | EU | cohort | EPIC, a prospective cohort with 23 centers in 10 European countries (Denmark, France, Germany, Greece, Italy, The Netherlands, Norway, Spain, Sweden, and the United Kingdom). Participants were aged 35 to 70 years at enrolment between 1992 and 2000. | Incident prostate cancer cases were identified through linkage to population cancer registries in Denmark, Italy, The Netherlands, Spain, Sweden, and the United Kingdom, or health insurance records, regional health departments, municipality registries, hospital- or physician-based cancer and pathology registries, and active follow-up through mail or phone calls to study participants or their next of kin in Germany and Greece. | | Exposure= 57.5±8.3 Control= 51.4**±**10.1 | | | Adjusted for education, smoking status, body mass index, waist circumference, and physical activity. |
| Jocelyn S. Kasper et al.^53^ | 2009 | US | cohort | The HPFS, an ongoing prospective cohort study of the causes of cancer and heart disease in men, consists of 51,529 United States male dentists, optometrists, osteopaths, podiatrists, pharmacists, and veterinarians, who were 40 to 75 years old at baseline | Participants were also asked to report new diagnoses of various cancers including that of the prostate. For each new report of prostate cancer, permission was requested from all study participants (or next-of-kin in the event of death) to obtain hospital and pathology reports, which were reviewed by blinded study investigators. | | Exposrue= 54.9 Control= 53.9 | | | Covariates include age, level of physical activity, body mass index at baseline and at 21 years old, height, ancestry, smoking habits, family history, and intakes of calories, bacon, tomato sauce, alpha-linolenic acid, calcium, fish, and vitamin E supplementation. |
| David J. Rosenberg et al.^54^ | 2002 | US | cohort | A hospital-based case-control study in our University Medical Center in New York City. | Cases were defined as all patients with incident histologically-diagnosed adenocarcinoma of the prostate seen at CPMC between January 1, 1984 and December 31, 1986, as identified by the CPMC Tumor Registry | | Case= 69.6±9.1 Control= 68.1±9.0 | | | Adjusted for age, race and CHD. |
| A. Tavani et al.^55^ | 2005 | Italy | case-control | A hospital-based case-control study conducted in Italy between 1991 and 2002 | Incident histologically confirmed prostate cancer | | NR | | | Estimates from multiple logistic regression models including terms for age, centre, education, body mass index, calorie intake, smoking and family history of prostate cancer. |
| Céline Lavalette et al.^56^ | 2022 | France | case-control | Based on data from the Epidemiological study of PCa (EPICAP), a population-based case-control study that included 819 incident PCa cases in 2012-2013 and 879 controls frequency matched by age) | From patient’s medical records and validated by the Hérault Cancer Registry | | Reported | | | NR |
| Clara Lam et al.^57^ | 2018 | US | case-control | The Surveillance, Epidemiology, and End Results (SEER)-Medicare linkage provided data on cancer-specific deaths and diabetes prevalence among 14 separate cohorts representing 1 068 098 cancer patients ages 66 + years diagnosed between 2000 and 2011 in 17 SEER areas | Identified through SEER‐Medicare data | | More than 66 | | | Adjusted for age, stage, comorbidities, and treatment. |
| Meng-Bo Hu et al.^58^ | 2019 | China | case-control | A case-control study was conducted among a prospectively enrolled prostate biopsy cohort of 518 patients from Jan 2013 to Dec 2014 at Huashan Hospital | A cohort of 559 consecutive patients who underwent initial multicore (≥10) prostate biopsy with transrectal ultrasound (TRUS) guidance from Jan 2013 to Dec 2014 | | Exposure= 72±13 Control= 70±13 | | | Adjusted for age, PSA, PV, DRE, and history of hypertension (PSA and PV were logarithmically transformed). |
| Aurora Perez-Cornago et al.^59^ | 2020 | British | cohort | UK Biobank, a prospective study of >500,000 people (aged 40–69 years, including 229,000 men) designed to be a resource for research into the causes of disease in middle and old age | Cancer incidence and mortality data were provided by the NHS Digital for England and Wales and by the NHS Central Register and Information and Statistics Division for Scotland. | | 56.53±8.20 | | | Adjusted for age (underlying time variable), Townsend deprivation score, ethnicity, lives with a wife or partner, BMI, smoking, physical activity, alcohol consumption and diabetes |
| Visalini Nair-Shalliker et al.^60^ | 2022 | Australia | cohort | The Sax Institute’s 45 and Up Study is a large NSW population-based cohort study of male and female participants aged 45 years and above. All participants were enrolled between 2006 and 2009. Participants were randomly sampled from Services Australia | From the linked records of Centre for Health Record Linkage (CHeReL) | | 61.0(45.0-102.8) | | | Adjusted for age (as underlying time variable), region of birth, health cover, income, qualification, place of residence, marital status, Charlson’s comorbidity index unless variable is the exposure of interest, frequency of PSA testing and frequency of GP visits. |
| Teemu J. Murtola et al.^61^ | 2018 | Finnish | cohort | Finnish Randomized Study of Screening for Prostate Cancer (FinRSPC) | Screened with PSA at four-year intervals (the screening arm, 31,866 men) or to control arm with no intervention and followed through national registries | | NR | | | Adjusted with age, the FinRSPC study arm and use of antihypertensive drugs, statins, NSAIDs or aspirin |
| Jeanne A. Darbinian et al.^62^ | 2008 | US | cohort | The men who were members of Kaiser Permanente Medical Care Program and had completed at least one Multiphasic Health Checkup (MHC) between July 1964 and August 1973 | Subjects were followed for development of prostate cancer using the Kaiser Permanente tumor registry computer-stored hospitalization records. | | 72(45-97) | | | NR |
| K. Zhu et al.^63^ | 2004 | US | case-control | Physicians’ Health Study, a completed randomized trial of aspirin and beta-carotene among 22,071 US male physicians aged 40–84 years conducted from 1982 to 1995 | Prostate cancer was self-reported by subjects, then confirmed by medical record review conducted by an endpoints committee. | | Reported | | | Adjusted for aspirin/beta-carotene assignment and race |
| Brook A. Calton et al.^64^ | 2007 | US | cohort | 328,316 men enrolled in the NIH-AARP Diet and Health Study. Participants were ages 50-71 years and without a prostate cancer diagnosis at baseline in 1995 | Identified by probabilistic linkage to eight state cancer registries. | | Exposure= 63.0 Control= 62.1 | | | The multivariable model was adjusted for current age (2.5-year categories), body mass index at baseline (<18.5, 18.5–21.9, 22.0–24.9, 25.0–27.4, 27.5–29.9, 30.0–34.9, 35.0–39.9, ≥40.0 kg/m2), height (<1.72, 1.72–1.75, 1.76–1.79, 1.80–1.83, 1.84–1.87, ≥1.88 m), education (<12 years, 12 years, post-high school or some college, college graduate, post graduate), race (White, Black, Hispanic, and a combined group of Asian, Pacific Islander, American Indian, and Alaskan Native), family history of prostate cancer (yes, no), smoking status (never smoking, currently smoking 1–10, 11–20, 21–30, 31–40, 41–60, ≥60 cigarettes per day, formerly smoking 1–10, 11–20, 21–30, 31–40, 41–60, ≥60 cigarettes per day), vigorous physical activity that lasted at least 20 min (never or rarely, 1–3 times/month, 1–2 times/week, 3–4 times/week, 5 or more times/week), supplemental vitamin E use (yes, no), supplemental zinc use (yes, no), alcohol intake (quintiles), and quintiles of energy-adjusted intakes of red meat, alpha-linolenic acid, tomato products, fish, calcium (from the combination of food and supplements), and vitamin D (from the combination of food and supplements) bIncludes regionally invasive cases of prostate cancer and prostate cancer deaths |
| J. C. Will et al.^65^ | 1999 | US | cohort | 1959–1972 Cancer Prevention Study, a 13-year follow-up period | Incident cases of prostate cancer were those revealed by any of the four follow-up questionnaires or the death certificate | | More than 30 | | | Adjusted by age |
| Jesús Gibran Hernández-Pérez et al.^66^ | 2022 | Mexico | case-control | Information from 394 incident PC-cases and 793 population age-matched (± 5 years) controls, identified in Mexico City (2011-2014) | Histologically confirmed, who were identified at the urology department of four third level and two second-level public hospitals. | | Case= 67.69±8.39 Control= 66.94±8.94 | | | Adjusted by age at interview, birthplace, education, family history of first-degree PC, history of sexually transmitted diseases, energy-adjusted dietary inflammatory index, life-course physical activity and smoking patterns; |
| Zhihong Gong et al.^67^ | 2006 | US | case-control | All data for this study were collected as part of the PCPT, a randomized, placebo-controlled trial testing. A total of 18,880 men ages ≥55 years with a normal digital rectal exam and prostate-specific antigen (PSA) level of ≤3 ng/mL | Men underwent digital rectal exam and PSA measures annually, and a prostate biopsy was recommended for participants with an abnormal digital rectal exam or a PSA of ≥4.0 ng/mL. | | Case= 63.7±5.6 Control= 62.6±5.4 | | | Adjusted for age, race, treatment, family history of prostate cancer in first-degree relatives, and BMI. |
| Gaurav Aggarwal et al.^68^ | 2013 | US | case-control | Identified consecutive patients who were diagnosed with lung cancer, breast cancer, prostate cancer, and colorectal cancer, at Mayo Clinic, Rochester in 2007 | Diagnosed in Mayo Clinic, Rochester in 2007. | | Case= 71.3+9.0 | | | NR |
| Rocío Barrios-Rodríguez et al.^69^ | 2022 | Spain | case-control | 1047 incident PCa cases and 1379 randomly selected controls, recruited in 7 Spanish provinces for the population-based MCC-Spain case. | PCa was classified according International Society of Urological Pathology (ISUP). Ten PCa cases could not be classified due to lack of information. | | Case= 65.9±7.4 Conrol= 66.4±8.6 | | | Adjusted for age, education level (no studies-primary/secondary/high school), body mass index (normal/overweight versus obesity), and family history of prostate cancer  (none/second-degree/first-degree). |
| C. C. Hsieh et al.^70^ | 1999 | Greece | case-control | From February 1994 to January 1997, 372 patients with histologically confirmed, newly diagnosed PC (incident cases) and residents of the Greater Athens area were contacted in any of 6 large hospitals of this area. | 52 were histologically identified following an operation for benign prostatic hyperplasia (BPH) | | Reported | | | Adjusted for age, height, body mass index and years of schooling. |
| Saima Shakil Malik et al.^71^ | 2018 | Pakistan | case-control | This study was conducted in 5 major hospitals in Pakistan including Nuclear medicine Oncology and Radiotherapy Institute (NORI) Islamabad, Shifa International Hospital Islamabad, Benazir Bhutto Shaheed Hospital Rawalpindi (BBH), Institute of Nuclear Medicine Oncology Lahore (INMOL), and Pakistan Navy Ship Shifa (PNS Shifa) Karachi. | Histopathologically confirmed | | Case= 70.25±8.02 Control=66.27±9.6 | | | NA |
| B. Ganesh et al.^72^ | 2011 | India | case-control | This is a retrospective unmatched hospital-based case-control study conducted at Tata Memorial Hospital that included subjects registered between the years 1999 and 2001. | Histologically confirmed | | Case= 64 Control= 46 | | | Adjusted for age, religion, education. |
| Elizabeth A. Atchison et al.^73^ | 2011 | US | cohort | The study cohort was identified using hospital discharge records dated between July 1, 1969 and September 30, 1996 from 142 nationwide United States Veterans Affairs (VA) hospitals. | Hospital discharge records | | Exposure= 57.5 Control= 51.5 | | | Adjusted for age, time, latency, race and number of visits;factors above and diagnoses of alcohol-related conditions, obesity and chronic obstructive pulmonary disease |
| Shih-Yi Lin et al.^74^ | 2021 | China(TW) | case-control | The National Health Insurance (NHI) program, a compulsory insurance program in Taiwan. | Men diagnosed with prostate cancer (ICD-9-CM code 185 with a major illness or injury certificate) between 2000 and 2012 were included in the case cohort. | | Case= 73.6 ± 9.11 Control= 73.6 ± 9.70 | | | multivariable analysis including age, occupation, urbanisation, insured monthly income (NTD), comorbidities and medications |
| K. A. Moses et al.^75^ | 2012 | US | case-control | A retrospective review of 3162 consecutive men who underwent prostate biopsy between January 2000 and July 2009 at the Atlanta Veterans Affairs Medical Center was performed. | All consecutive patients who underwent transrectal ultrasound-guided prostate biopsy for elevated PSA and/or digital rectal examination (DRE) | | Reported | | | Controlled for age, race, body mass index, prostate volume, family history of prostate cancer, PSA and DRE results, and interaction between PSA and DRE |
| Birgitta Grundmark et al.^76^ | 2010 | Sweden | cohort | In the prospective Uppsala Longitudinal Study of Adult Men (ULSAM) of 2322 Caucasian men with 34 years of follow-up baseline MetS-measurements at age 50 were used. | Identified using national registries medical records. | | Case= 73(51-83) | | | NR |
| N. Baradaran et al.^77^ | 2009 | Iran | case-control | Using data from a multi-center case-control study in Iran | In brief, cases were 194 men with incident, clinicopathologically confirmed PC, admitted to the major teaching hospitals in the provinces under surveillance of eight large universities of Iran. | | Case= 71.06±7.8 Control= 66.5±10.2 | | | Adjusted by age |
| Eric A. Miller et al.^78^ | 2018 | US | cohort | The PLCO trial included men and women aged 55–74 years with no previous PLCO cancer or current cancer treatment who were enrolled between 1993 and 2001 from 10 cancer centers across the U.S. |  | | | Exposure= 63.9 Control= 62.6 | Adjusted by age | |
| E. L. Turner et al.^79^ | 2011 | British | case-control | In ProtecT, men aged 50–69 years in 206 general practices located around 9 UK cities. | Men with a raised PSA (≥3 ng/ml) were invited for digital rectal examination and transrectal ultrasound-guided biopsy (10 cores) | | Case= 62.2±5.0 Control= 62.0±4.9 | | | Adjusted for age. |
| Rachael Williams et al.^80^ | 2018 | British | case-control | CPRD primary care, HES APC, cancer registry and death certificate data. | International Classification of Diseases 10th Revision (ICD-10) codes were used to identify cancer across HES APC, cancer registry and death certificate data (with ICD-9 being used for deaths prior to 2001), with diagnoses in primary care being made and identified using Read codes | | More than 40 | | | Adjusted for age, sex, year of start of follow-up, smoking status, use of alcohol, body mass index, prescribing in the 6 months prior to the start of follow-up (angiotensin II receptor blockers, antiplatelets, beta blockers, calcium channel blockers, diuretics, nitrates, NSAIDs or aspirin and statins), Index of Multiple Deprivation and medical history (coronary heart disease, coronary revascularisation, hyperlipidaemia, hypertension, peripheral vascular disease, renal impairment and stable angina). |
| Cristina Bosetti et al.^81^ | 2012 | Italy and Switherland | case-control | An integrated series of case-control studies conducted in Italy and Switzerland between 1991 and 2009 | Identified in the major teaching and general hospitals of the study areas. | | Case= 66 Control= 63 | | | Adjusted for sex (when appropriate), age, study center, year of interview, education, alcohol drinking, and tobacco smoking and body mass index. |
| Hadith Rastad et al.^82^ | 2019 | US | cohort | The data from the Atherosclerosis Risk in Communities (ARIC), an ongoing multicenter prospective cohort study in four U.S. communities, were used in this study. | A diagnosis of cancer (outcome), the cancer site, and the date of diagnosis were determined based on participants’ self-reported data obtained through interviews at baseline and follow-up visits. | | 45-64 | | | Adjusted for age, Body Mass Index, Physical activity and alcohol consumption using stratified poisson regression; |
| Marie-Claude Rousseau et al.^83^ | 2006 | Canada | case-control | A large multicancer case-control project carried out in Montreal, Canada, in the 1980s. This report, based on 3,107 male cancer cases and 509 population controls, uses information on diabetes and several covariates collected by interview. | Cases were ascertained from the major hospitals in Montreal, providing almost complete coverage (97%) of all incident cancer cases diagnosed between 1979 and 1985 | | Case= 62.9±5.0 Control= 59.6±7.9 | | | Adjusted for age, family income, years of schooling, ethnicity, proxy status, body mass index. And tobacco, acohol and farming |
| Kozue Nakamura et al.^84^ | 2013 | Japan | cohort | Participants in the present study were from the Takayama Study cohort. In September 1992, that population-based prospective study was initiated among 36 990 residents of Takayama, Gifu Prefecture, Japan, who were over 35 years of age. | Cancer occurrence was mostly ascertained by means of a linkage with the records of two local population-based cancer registries. | | Exposure= 59.3±11.2 Control=54.3±12.3 | | | Adjusted for age at baseline, smoking status, body mass index, physical activity, length of education in years, history of hypertension, history of stroke, history of ischemic heart disease, total energy intake, and intake of fat, ethanol, and coffee. CI, confidence interval. |
| Jennifer L. Beebe-Dimmer et al.^85^ | 2007 | US | case-control | Data collection for the FMHS began in 1996 and concluded in 2002. Informed consent was obtained from study participants, and all protocols were approved by the Institutional Review Board at the University of Michigan Medical School. African-American men between ages 40 years and 79 years were identified from a probability sample with over sampling of men in older age groups. | Men who had an abnormal digital rectal examination and/or elevated total PSA concentration (≥4.0 ng/mL) were referred for prostate biopsy. Twenty men who subsequently were diagnosed with biopsy-confirmed prostate cancer were included in the study as cases | | Case= 67.4±8.8 Control62.1±10.1 | | | Adjusted for age and smoking history. |
| Qiang Li et al.^37^ | 2010 | Japan | case-control | The authors examined the relationship between diabetes mellitus and the risk of prostate cancer in the Ohsaki cohort followed from 1995 to 2003, in which 230 new cases of prostate cancer were identified among 22,458 Japanese men | Through a computerized record linkage to the Miyagi Prefecture Cancer Registry. Ascertained the death, emigration, or loss of NHI qualification of each of the study subjects by obtaining their NHI withdrawal history files from the Miyagi NHI Association. | | Exposure= 62.41±9.34 Control= 59.07±10.62 | | | Adjusted for age (continuous variable), family history of cancer (yes or no), BMI (<18.5, 18.5–24.9, or ≥25.0, calculated as weight in kilograms divided by height in meters squared), smoking status (never, former, currently smoking <20 cigarettes/day, or currently smoking ≥20 cigarettes/day), total energy intake per day (continuous variable) and average sleep duration (≤6, 7–8, or ≥9 h/day) |
| Sui-Foon Lo et al.^86^ | 2013 | China(TW) | case-control | The single-payer NHI Program in Taiwan that began in 1995 consolidated 13 insurance programs into a national system to provide healthcare for all residents. | The subjects were linked to the registry of Catastrophic Illness Patient Database (CIPD) to identify those who had the diagnosis of cancer in the NHI program through histologic confirmation. | | Exposure= 60.5±13.6 Control= 60.4±13.7 | | | Adjusted for age, sex, urbanization, hypertension and hyperlipidemia |
| Wallstrom P et al.^87^ | 2009 | Sweden | case-control | Malmö Diet and Cancer (MDC) study, in Sweden's third largest city, consists of all men born in 1923–1945 and all women born in 1923–1950 who were living in Malmö during the screening period 1991–1996 (n=74 138). | Cancer cases were ascertained by record linkage with the National Cancer Register. | | NR | | | Adjusted for age, height, co-habitation status, socioeconomic status, alcohol habits, smoking habits, prevalent diabetes, total physical activity, birth country, and total intake of EPA, DHA, red meat, and calcium. Height and prevalent diabetes were further adjusted for BMI category. |
| Strom S.S et al.^88^ | 2008 | US | case-control | This population-based case-control study included 176 Texas men of Mexican descent with PCa and 174 age- and ethnicity-matched controls. | The 268 patients identified through medical records | | Case= 62.2±8.0 Control= 62.1±6.7 | | | Adjusted for age, education, screening history, and First-degree family history of PCa, Agrichemical exposure, Occupational physical activity |
| Gholamreza Pourmand et al.^89^ | 2007 | Iran | case-control | The data were derived from a case-control study of PC, conducted prospectively between August 2005 and May 2007 in eight provinces of Iran: Tehran, Isfahan [Central Iran], Hamadan, Lorestan | Cases were 130 men with incident, clinicopathologically confirmed PC, admitted to the major referral and teaching hospitals in the areas under surveillance | | Case= 70.55±8.28 Control= 65.66±9.87 | | | NR |
| Hsin-Chieh Yeh et al.^90^ | 2012 | US | case-control | Prospective data on 599 diabetic and 17,681 nondiabetic adults from the CLUE II (Give Us a Clue to Cancer and Heart Disease) cohort in Washington County, Maryland, were analyzed. | Identified through linkage of the cohort participants with the Washington County Cancer Registry and, since 1992, with the Maryland Cancer Registry. | | Exposure= 61.8±11.2 Control= 51.5±13.7 | | | Adjusted for age, the square of age, sex, BM1, smoking, educatior evel. hypertension treatmen. and high cholesterol treatmen |
| Chaoyang Li et al.^91^ | 2011 | US | cohort | Data for 397,783 adults who participated in the 2009 Behavioral Risk Factor Surveillance System and had valid data on diabetes and cancer | Cancer status was ascertained by asking participants. | | 46.8(18-99) | | | Adjusted for age (years, continuous), race/ethnicity (non–Hispanic white, non–Hispanic black, Hispanic, other), health insurance coverage (any vs. none), smoking status (current smoker, former smoker, never smoked), heavy drinking (yes, consuming more than two drinks per day among men and more than one drink per day among women vs. no), BMI (kg/m2, continuous), and physical inactivity (yes vs. no). |
| Sheng-Hwu Hsieh et al.^38^ | 2014 | China(TW) | cohort | Chang Gung Memorial Hospital, Linkou, Taiwan, from January 2000 to December 2010. | The patients were identified through hospital admission data at Chang Gung Memorial Hospital, Linkou, Taiwan, from January 2000 to December 2010. Patients 20 years or older were included if the reason for their hospital admission was malignant neoplasm as indicated by International Classification of Diseases, Ninth Revision (ICD-9), codes 140 to 208.91. | | Reported | | | NR |
| Bo Attner et al.^92^ | 2012 | Sweden | cohort | All patients with cancer diagnoses from 2005–2007 were identified in the Cancer Register for Southern Sweden. | All patients with cancer diagnoses from 2005–2007 were identified in the Cancer Register for Southern Sweden | | NR | | | NR |
| H. O. Adami et al.^93^ | 1991 | Sweden | cohort | From 1965 through 1983, the Swedish National Board of Health and Welfare received annual reports from all inpatient medical institutions in Sweden and recorded data on individual hospital admissions and discharges in the Inpatient Register. | These diagnoses were coded according to the seventh revision of the International Classification of Diseases through 1968, and according to the eighth revision there after. | | More than 20 | | | NR |
| Richard M. Martin et al.^94^ | 2009 | Norway | cohort | Between 1995 and 1997, all residents in Nord-Trøndelag County in Norway aged 20 years or older were invited to participate in the second wave of the Nord-Trøndelag Health Study (HUNT 2). | The unique 11-digit identity number of Norwegian citizens was used to link individuals from the HUNT Study to information on cancer incidence at the Cancer Registry of Norway. Prostate cancer was registered according to the International Classification of Diseases, seventh edition (ICD-7, code 177). | | Exposure= 48.0±16.4 Control= 53.4±16.3 | | | Adjusted for age, height (quarters), smoking (never, former, current, and unknown), marital status (married, unmarried, widower, and divorced/separated), education (<10, 10–12, and ≥13 years), physical activity (no activity, <3 h light, ≥3 h light or <1 h hard, ≥1 h hard, and unknown), International Prostate Symptom Score (none, mild, moderate, and severe lower urinary tract symptoms); and blood pressure measures also adjusted for the use of blood pressure medication |
| C. Wu et al.^95^ | 2011 | Worldwide | cohort | Reduction by Dutasteride of Prostate Cancer Events (REDUCE) trial | Serum PSA was measured every 6 months. Prostate volume was determined by transrectal ultrasound at time of randomization. Repeat 10-core biopsies were obtained at 2 and 4 years regardless of PSA or digital rectal examination findings. | | Exposure= 64(59–68) Control= 63(58–67) | | | Abbreviations: BMI, body mass index; DRE, digital rectal examination;REDUCE, Reduction by Dutasteride of Prostate Cancer Events |
| Cuiping Bao et al.^96^ | 2018 | Sweden | cross-section | Participants were members of the nationwide Swedish Twin Registry (STR), which was started in the 1960s. | Cancer diagnoses were derived from the NPR and the Swedish Cancer Registry (SCR) between 1998 and 2014. The site-specific cancer forms, based on ICD code. | | Case= 65.1±7.3 Control= 60.0±7.8 | | | Adjusted for age, education, body mass index, smoking, binge drinking and marital status. |
| Dianna J. Magliano et al.^97^ | 2012 | Australia | cohort | The Fremantle Diabetes Study (FDS) Phase I was a longitudinal observational cohort study of patients from a postcode-defined urban community of 120 097 people. | All deaths, hospitalisations and cancer registrations in the state of Western Australia are recorded in the Western Australia Data Linkage System | | Case= 65.2±9.2 Control= 63.1±12.0 | | | NR |
| Lourdes Guerrios-Rivera et al.^98^ | 2023 | US | cross-section | Men at Durham Veterans Affairs Hospital | Eligible subjects were men with no prior PC history undergoing a prostate biopsy because of abnormal PSA and/or suspicious digital rectal exam (DRE) as clinically indicated. For men with multiple biopsies performed at the DVAMC, only used data from their initial biopsy at the DVAMC | | Exposure= 64 Control= 63 | | | Adjusted for age, year, race, log-transformed PSA, DRE, and log-transformed volume |
| A. González-Pérez et al.^99^ | 2005 | UK | case-control | All males 50–79 years old between January 1995 and December 2001. Patients with a code for any cancer before starting date were excluded. | Reviewed the computerized patient profiles. | | 50–79 | | | Adjusted for age, calendar year, NSAID use, prior history of prostatism, body mass index, health care utilization (GP visits, referrals, hospitalizations), and all the variables included in the table using unconditional logistic regression. |
| Lauren P. Wallner et al.^100^ | 2011 | US | cohort | In 1990, a randomly selected cohort of Caucasian men from Olmsted County, MN, USA, aged 40-79 years, was recruited; 2445 completed a questionnaire that included physician-diagnosed diabetes and hypertension | Biopsy-confirmed cases of prostate cancer were identified through detailed review of medical records | | More than 40 | | | NR |
| Bendix Carstensen et al.^101^ | 2016 | Australia, Denmark, Finland, Scotland and Sweden | cohort | Persons with type 1 diabetes were identified from five nationwide diabetes registers: Australia (2000–2008), Denmark (1995–2014), Finland (1972–2012), Scotland (1995–2012) and Sweden (1987–2012) | Linkage to national cancer registries provided the numbers of incident cancers in people with type 1 diabetes and in the general population | | NR | | | NR |
| Bjornsdottir H.H. et al. ^102^ | 2018 | Sweden | case-control | Included patients defined by the epidemiological definition as T2DM in the Swedish National Diabetes Register (NDR） | Linkage to nation cancer registeries | NR | | | | NR |
| Karlin N.J. et al.^103^ | 2023 | US | cross-section | The cancer center at the institution encompasses academic campuses in the Southeast, Southwest and Midwest (the sites included in this analysis). It is a comprehensive cancer center designated by the National Cancer Institute, with coordinated care and collaboration across the three sites. | Cases were identified by pathology results and by International Classification of Diseases, Ninth Revision codes | | Exposure= 67±10 Control= 67±10 | | | NR |
| Mingyang Song et al.^104^ | 2020 | US | cohort | Followed for cancer incidence 113 429 women in the Nurses’ Health Study (1978-2014) and 45 604 men in the Health Professionals Follow-up Study (1988-2014) who were free of diabetes and cancer at baseline | Cancer diagnosis was confirmed by medical record review among participants who reported a diagnosis of cancer on the follow-up questionnaires. | | Exposure= 68.0±9.6 Control= 58.7±11.9 | | | Adjusting for age (month), ethnicity (White, African American, Asian, others), smoking status (never smoked, past smoker, currently smoke 1-14 cigarettes per day, 15-24 cigarettes per day, or ≥25 cigarettes per day), alcohol intake (0, 0.1-4.9, 5.0-9.9, 10.0-14.9, 15.0-29.9, and ≥30.0 g/d), multivitamin use (yes, no), physical activity (quintiles), total energy (quintiles), alternative healthy eating index (quintiles), family history of diabetes (yes, no), family history of cancer (yes, no), endoscopy screening (yes, no), and fasting glucose screening (yes, no). For women, insulin use (yes, no), oral hypoglycemic drug use (yes, no), mammography screening, postmenopausal hormone use (never, former, or current hormone use, or missing), and oral contraceptive use were further adjusted |
| Chen C.B. et al.^105^ | 2018 | Canada | case-control | Using administrative data from British Columbia, Canada for the period 1994 to 2012, we identified men aged ≥50 years with and without diabetes. | Eliminating cases of cancer within the 2 years prior to the index date, cases of prostate cancer were identified (ICD-O-3 code C61) from the British Columbia Cancer Agency | | Exposure= 64.7±9.4 Control= 64.7±9.4 | | | NR |
| Walker J.J. et al.^106^ | 2013 | UK | cohort | Data for people diagnosed with diabetes in Scotland were obtained from the Scottish Care Information – Diabetes Collaboration (SCI-DC) dataset. This holds clinical and demographic data on almost all patients with diabetes in Scotland | The objective of this study was to use Scottish national data. All people in Scotland aged 55-79 years diagnosed with any of the cancers of interest during the period 2001-2007 were identified. | | 55-79 | | | Adjusted for age and socioeconomic status |

References

38. La Vecchia C, Negri E, Franceschi S, D’Avanzo B, Boyle P. A case-control study of diabetes mellitus and cancer risk. Br J Cancer. 1994; 70:950–3.

<https://doi.org/10.1038/bjc.1994.427> PMID:[7947103](https://pubmed.ncbi.nlm.nih.gov/7947103)

39. Tindall EA, Bornman MS, van Zyl S, Segone AM, Monare LR, Venter PA, Hayes VM. Addressing the contribution of previously described genetic and epidemiological risk factors associated with increased prostate cancer risk and aggressive disease within men from South Africa. BMC Urol. 2013; 13:74.

<https://doi.org/10.1186/1471-2490-13-74> PMID:[24373635](https://pubmed.ncbi.nlm.nih.gov/24373635)

40. Suarez Arbelaez MC, Nackeeran S, Shah K, Blachman-Braun R, Bronson I, Towe M, Bhat A, Marcovich R, Ramasamy R, Shah HN. Association between body mass index, metabolic syndrome and common urologic conditions: a cross-sectional study using a large multi-institutional database from the United States. Ann Med. 2023; 55:2197293.

<https://doi.org/10.1080/07853890.2023.2197293> PMID:[37036830](https://pubmed.ncbi.nlm.nih.gov/37036830)

41. Lawrence YR, Morag O, Benderly M, Boyko V, Novikov I, Dicker AP, Goldbourt U, Behar S, Barchana M, Wolf I. Association between metabolic syndrome, diabetes mellitus and prostate cancer risk. Prostate Cancer Prostatic Dis. 2013; 16:181–6.

<https://doi.org/10.1038/pcan.2012.54> PMID:[23399780](https://pubmed.ncbi.nlm.nih.gov/23399780)

42. Waters KM, Henderson BE, Stram DO, Wan P, Kolonel LN, Haiman CA. Association of diabetes with prostate cancer risk in the multiethnic cohort. Am J Epidemiol. 2009; 169:937–45.

<https://doi.org/10.1093/aje/kwp003> PMID:[19240222](https://pubmed.ncbi.nlm.nih.gov/19240222)

43. Lin E, Garmo H, Van Hemelrijck M, Adolfsson J, Stattin P, Zethelius B, Crawley D. Association of type 2 diabetes mellitus and antidiabetic medication with risk of prostate cancer: a population-based case-control study. BMC Cancer. 2020; 20:551.

<https://doi.org/10.1186/s12885-020-07036-4> PMID:[32539807](https://pubmed.ncbi.nlm.nih.gov/32539807)

44. Ballotari P, Vicentini M, Manicardi V, Gallo M, Chiatamone Ranieri S, Greci M, Giorgi Rossi P. Diabetes and risk of cancer incidence: results from a population-based cohort study in northern Italy. BMC Cancer. 2017; 17:703.

<https://doi.org/10.1186/s12885-017-3696-4> PMID:[29070034](https://pubmed.ncbi.nlm.nih.gov/29070034)

45. Chodick G, Heymann AD, Rosenmann L, Green MS, Flash S, Porath A, Kokia E, Shalev V. Diabetes and risk of incident cancer: a large population-based cohort study in Israel. Cancer Causes Control. 2010; 21:879–87.

<https://doi.org/10.1007/s10552-010-9515-8> PMID:[20148361](https://pubmed.ncbi.nlm.nih.gov/20148361)

46. Rodriguez C, Patel AV, Mondul AM, Jacobs EJ, Thun MJ, Calle EE. Diabetes and risk of prostate cancer in a prospective cohort of US men. Am J Epidemiol. 2005; 161:147–52.

<https://doi.org/10.1093/aje/kwh334> PMID:[15632264](https://pubmed.ncbi.nlm.nih.gov/15632264)

47. Tavani A, Gallus S, Bosetti C, Tzonou A, Lagiou P, Negri E, Trichopoulos D, La Vecchia C. Diabetes and the risk of prostate cancer. Eur J Cancer Prev. 2002; 11:125–8.

<https://doi.org/10.1097/00008469-200204000-00003> PMID:[11984129](https://pubmed.ncbi.nlm.nih.gov/11984129)

48. Velicer CM, Dublin S, White E. Diabetes and the risk of prostate cancer: the role of diabetes treatment and complications. Prostate Cancer Prostatic Dis. 2007; 10:46–51.

<https://doi.org/10.1038/sj.pcan.4500914> PMID:[17033617](https://pubmed.ncbi.nlm.nih.gov/17033617)

49. Pierce BL, Plymate S, Ostrander EA, Stanford JL. Diabetes mellitus and prostate cancer risk. Prostate. 2008; 68:1126–32.

<https://doi.org/10.1002/pros.20777> PMID:[18459104](https://pubmed.ncbi.nlm.nih.gov/18459104)

50. Coker AL, Sanderson M, Zheng W, Fadden MK. Diabetes mellitus and prostate cancer risk among older men: population-based case-control study. Br J Cancer. 2004; 90:2171–5.

<https://doi.org/10.1038/sj.bjc.6601857> PMID:[15150583](https://pubmed.ncbi.nlm.nih.gov/15150583)

51. Leitzmann MF, Ahn J, Albanes D, Hsing AW, Schatzkin A, Chang SC, Huang WY, Weiss JM, Danforth KN, Grubb RL 3rd, Andriole GL, and Prostate, Lung, Colorectal, and Ovarian Trial Project Team. Diabetes mellitus and prostate cancer risk in the Prostate, Lung, Colorectal, and Ovarian Cancer Screening Trial. Cancer Causes Control. 2008; 19:1267–76.

<https://doi.org/10.1007/s10552-008-9198-6> PMID:[18618278](https://pubmed.ncbi.nlm.nih.gov/18618278)

52. Tsilidis KK, Allen NE, Appleby PN, Rohrmann S, Nöthlings U, Arriola L, Gunter MJ, Chajes V, Rinaldi S, Romieu I, Murphy N, Riboli E, Tzoulaki I, et al. Diabetes mellitus and risk of prostate cancer in the European Prospective Investigation into Cancer and Nutrition. Int J Cancer. 2015; 136:372–81.

<https://doi.org/10.1002/ijc.28989> PMID:[24862312](https://pubmed.ncbi.nlm.nih.gov/24862312)

53. Kasper JS, Liu Y, Giovannucci E. Diabetes mellitus and risk of prostate cancer in the health professionals follow-up study. Int J Cancer. 2009; 124:1398–403.

<https://doi.org/10.1002/ijc.24044> PMID:[19058180](https://pubmed.ncbi.nlm.nih.gov/19058180)

54. Rosenberg DJ, Neugut AI, Ahsan H, Shea S. Diabetes mellitus and the risk of prostate cancer. Cancer Invest. 2002; 20:157–65.

<https://doi.org/10.1081/cnv-120001141> PMID:[11901534](https://pubmed.ncbi.nlm.nih.gov/11901534)

55. Tavani A, Gallus S, Bertuzzi M, Dal Maso L, Zucchetto A, Negri E, Franceschi S, Ramazzotti V, Montella M, La Vecchia C. Diabetes mellitus and the risk of prostate cancer in Italy. Eur Urol. 2005; 47:313–7.

<https://doi.org/10.1016/j.eururo.2004.10.027> PMID:[15716192](https://pubmed.ncbi.nlm.nih.gov/15716192)

56. Lavalette C, Cordina-Duverger E, Rébillard X, Lamy PJ, Trétarre B, Cénée S, Menegaux F. Diabetes, metabolic syndrome and prostate cancer risk: Results from the EPICAP case-control study. Cancer Epidemiol. 2022; 81:102281.

<https://doi.org/10.1016/j.canep.2022.102281> PMID:[36279644](https://pubmed.ncbi.nlm.nih.gov/36279644)

57. Lam C, Cronin K, Ballard R, Mariotto A. Differences in cancer survival among white and black cancer patients by presence of diabetes mellitus: Estimations based on SEER-Medicare-linked data resource. Cancer Med. 2018; 7:3434–44.

<https://doi.org/10.1002/cam4.1554>
PMID:[29790667](https://pubmed.ncbi.nlm.nih.gov/29790667)

58. Hu MB, Bai PD, Wu YS, Zhang LM, Zhu WH, Hu JM, Yang T, Jiang HW, Ding Q. Effects of diabetes mellitus and Metformin administration on prostate cancer detection at biopsy among Chinese men: a case-control study. J BUON. 2019; 24:227–32.

PMID:[30941974](https://pubmed.ncbi.nlm.nih.gov/30941974)

59. Perez-Cornago A, Fensom GK, Andrews C, Watts EL, Allen NE, Martin RM, Van Hemelrijck M, Key TJ, Travis RC. Examination of potential novel biochemical factors in relation to prostate cancer incidence and mortality in UK Biobank. Br J Cancer. 2020; 123:1808–17.

<https://doi.org/10.1038/s41416-020-01081-3> PMID:[32963348](https://pubmed.ncbi.nlm.nih.gov/32963348)

60. Nair-Shalliker V, Bang A, Egger S, Yu XQ, Chiam K, Steinberg J, Patel MI, Banks E, O’Connell DL, Armstrong BK, Smith DP. Family history, obesity, urological factors and diabetic medications and their associations with risk of prostate cancer diagnosis in a large prospective study. Br J Cancer. 2022; 127:735–46.

<https://doi.org/10.1038/s41416-022-01827-1> PMID:[35610365](https://pubmed.ncbi.nlm.nih.gov/35610365)

61. Murtola TJ, Vihervuori VJ, Lahtela J, Talala K, Taari K, Tammela TL, Auvinen A. Fasting blood glucose, glycaemic control and prostate cancer risk in the Finnish Randomized Study of Screening for Prostate Cancer. Br J Cancer. 2018; 118:1248–54.

<https://doi.org/10.1038/s41416-018-0055-4> PMID:[29563633](https://pubmed.ncbi.nlm.nih.gov/29563633)

62. Darbinian JA, Ferrara AM, Van Den Eeden SK, Quesenberry CP Jr, Fireman B, Habel LA. Glycemic status and risk of prostate cancer. Cancer Epidemiol Biomarkers Prev. 2008; 17:628–35.

<https://doi.org/10.1158/1055-9965.EPI-07-2610> PMID:[18349280](https://pubmed.ncbi.nlm.nih.gov/18349280)

63. Zhu K, Lee IM, Sesso HD, Buring JE, Levine RS,
Gaziano JM. History of diabetes mellitus and risk of prostate cancer in physicians. Am J Epidemiol. 2004; 159:978–82.

<https://doi.org/10.1093/aje/kwh139>
PMID:[15128610](https://pubmed.ncbi.nlm.nih.gov/15128610)

64. Calton BA, Chang SC, Wright ME, Kipnis V, Lawson K, Thompson FE, Subar AF, Mouw T, Campbell DS, Hurwitz P, Hollenbeck A, Schatzkin A, Leitzmann MF. History of diabetes mellitus and subsequent prostate cancer risk in the NIH-AARP Diet and Health Study. Cancer Causes Control. 2007; 18:493–503.

<https://doi.org/10.1007/s10552-007-0126-y> PMID:[17450441](https://pubmed.ncbi.nlm.nih.gov/17450441)

65. Will JC, Vinicor F, Calle EE. Is diabetes mellitus associated with prostate cancer incidence and survival? Epidemiology. 1999; 10:313–8.

PMID:[10230844](https://pubmed.ncbi.nlm.nih.gov/10230844)

66. Hernández-Pérez JG, Torres-Sánchez L, Hernández-Alcaráz C, López-Carrillo L, Rodríguez-Covarrubias F, Vázquez-Salas RA, Galván-Portillo M. Metabolic Syndrome and Prostate Cancer Risk: A Population Case-control Study. Arch Med Res. 2022; 53:594–602.

<https://doi.org/10.1016/j.arcmed.2022.07.003> PMID:[35909002](https://pubmed.ncbi.nlm.nih.gov/35909002)

67. Gong Z, Neuhouser ML, Goodman PJ, Albanes D, Chi C, Hsing AW, Lippman SM, Platz EA, Pollak MN, Thompson IM, Kristal AR. Obesity, diabetes, and risk of prostate cancer: results from the prostate cancer prevention trial. Cancer Epidemiol Biomarkers Prev. 2006; 15:1977–83.

<https://doi.org/10.1158/1055-9965.EPI-06-0477> PMID:[17035408](https://pubmed.ncbi.nlm.nih.gov/17035408)

68. Aggarwal G, Kamada P, Chari ST. Prevalence of diabetes mellitus in pancreatic cancer compared to common cancers. Pancreas. 2013; 42:198–201.

<https://doi.org/10.1097/MPA.0b013e3182592c96> PMID:[23000893](https://pubmed.ncbi.nlm.nih.gov/23000893)

69. Barrios-Rodríguez R, García-Esquinas E, Pérez-Gómez B, Castaño-Vinyals G, Llorca J, de Larrea-Baz NF, Olmedo-Requena R, Vanaclocha-Espi M, Alguacil J, Fernández-Tardón G, Fernández-Navarro P, Cecchini L, Lope V, et al. Prostate cancer genetic propensity risk score may modify the association between this tumour and type 2 diabetes mellitus (MCC-Spain study). Prostate Cancer Prostatic Dis. 2022;
25:694–9.

<https://doi.org/10.1038/s41391-021-00446-w> PMID:[34601492](https://pubmed.ncbi.nlm.nih.gov/34601492)

70. Hsieh CC, Thanos A, Mitropoulos D, Deliveliotis C, Mantzoros CS, Trichopoulos D. Risk factors for prostate cancer: a case-control study in Greece. Int J Cancer. 1999; 80:699–703.

[https://doi.org/10.1002/(sici)1097-0215(19990301)80:5<699::aid-ijc12>3.0.co;2-7](https://doi.org/10.1002/(sici)1097-0215(19990301)80:5%3c699::aid-ijc12%3e3.0.co;2-7) PMID:[10048970](https://pubmed.ncbi.nlm.nih.gov/10048970)

71. Malik SS, Batool R, Masood N, Yasmin A. Risk factors for prostate cancer: A multifactorial case-control study. Curr Probl Cancer. 2018; 42:337–43.

<https://doi.org/10.1016/j.currproblcancer.2018.01.014> PMID:[29433825](https://pubmed.ncbi.nlm.nih.gov/29433825)

72. Ganesh B, Saoba SL, Sarade MN, Pinjari SV. Risk factors for prostate cancer: An hospital-based case-control study from Mumbai, India. Indian J Urol. 2011; 27:345–50.

<https://doi.org/10.4103/0970-1591.85438> PMID:[22022057](https://pubmed.ncbi.nlm.nih.gov/22022057)

73. Atchison EA, Gridley G, Carreon JD, Leitzmann MF, McGlynn KA. Risk of cancer in a large cohort of U.S. veterans with diabetes. Int J Cancer. 2011; 128:635–43.

<https://doi.org/10.1002/ijc.25362> PMID:[20473855](https://pubmed.ncbi.nlm.nih.gov/20473855)

74. Lin SY, Lin CL, Chang SS, Chang YH, Hsu WH, Lin CC, Kao CH. Risk of diabetes mellitus in patients with prostate cancer receiving injection therapy: A nationwide population-based propensity score-matched study. Int J Clin Pract. 2021; 75:e14416.

<https://doi.org/10.1111/ijcp.14416> PMID:[34047432](https://pubmed.ncbi.nlm.nih.gov/34047432)

75. Moses KA, Utuama OA, Goodman M, Issa MM. The association of diabetes and positive prostate biopsy in a US veteran population. Prostate Cancer Prostatic Dis. 2012; 15:70–4.

<https://doi.org/10.1038/pcan.2011.40> PMID:[21894176](https://pubmed.ncbi.nlm.nih.gov/21894176)

76. Grundmark B, Garmo H, Loda M, Busch C, Holmberg L, Zethelius B. The metabolic syndrome and the risk of prostate cancer under competing risks of death from other causes. Cancer Epidemiol Biomarkers Prev. 2010; 19:2088–96.

<https://doi.org/10.1158/1055-9965.EPI-10-0112> PMID:[20647401](https://pubmed.ncbi.nlm.nih.gov/20647401)

77. Baradaran N, Ahmadi H, Salem S, Lotfi M, Jahani Y, Baradaran N, Mehrsai AR, Pourmand G. The protective effect of diabetes mellitus against prostate cancer: role of sex hormones. Prostate. 2009; 69:1744–50.

<https://doi.org/10.1002/pros.21023> PMID:[19676082](https://pubmed.ncbi.nlm.nih.gov/19676082)

78. Miller EA, Pinsky PF, Pierre-Victor D. The relationship between diabetes, prostate-specific antigen screening tests, and prostate cancer. Cancer Causes Control. 2018; 29:907–14.

<https://doi.org/10.1007/s10552-018-1067-3> PMID:[30094676](https://pubmed.ncbi.nlm.nih.gov/30094676)

79. Turner EL, Lane JA, Donovan JL, Davis MJ, Metcalfe C, Neal DE, Hamdy FC, Martin RM. Association of diabetes mellitus with prostate cancer: nested case-control study (Prostate testing for cancer and treatment study). Int J Cancer. 2011; 128:440–6.

<https://doi.org/10.1002/ijc.25360> PMID:[20473853](https://pubmed.ncbi.nlm.nih.gov/20473853)

80. Williams R, van Staa TP, Gallagher AM, Hammad T, Leufkens HG, de Vries F. Cancer recording in patients with and without type 2 diabetes in the Clinical Practice Research Datalink primary care data and linked hospital admission data: a cohort study. BMJ Open. 2018; 8:e020827.

<https://doi.org/10.1136/bmjopen-2017-020827> PMID:[29804063](https://pubmed.ncbi.nlm.nih.gov/29804063)

81. Bosetti C, Rosato V, Polesel J, Levi F, Talamini R, Montella M, Negri E, Tavani A, Zucchetto A, Franceschi S, Corrao G, La Vecchia C. Diabetes mellitus and cancer risk in a network of case-control studies. Nutr Cancer. 2012; 64:643–51.

<https://doi.org/10.1080/01635581.2012.676141> PMID:[22519904](https://pubmed.ncbi.nlm.nih.gov/22519904)

82. Rastad H, Parsaeian M, Shirzad N, Mansournia MA, Yazdani K. Diabetes mellitus and cancer incidence: the Atherosclerosis Risk in Communities (ARIC) cohort study. J Diabetes Metab Disord. 2019; 18:65–72.

<https://doi.org/10.1007/s40200-019-00391-5> PMID:[31275876](https://pubmed.ncbi.nlm.nih.gov/31275876)

83. Rousseau MC, Parent ME, Pollak MN, Siemiatycki J. Diabetes mellitus and cancer risk in a population-based case-control study among men from Montreal, Canada. Int J Cancer. 2006; 118:2105–9.

<https://doi.org/10.1002/ijc.21600> PMID:[16284945](https://pubmed.ncbi.nlm.nih.gov/16284945)

84. Nakamura K, Wada K, Tamai Y, Tsuji M, Kawachi T, Hori A, Takeyama N, Tanabashi S, Matsushita S, Tokimitsu N, Nagata C. Diabetes mellitus and risk of cancer in Takayama: a population-based prospective cohort study in Japan. Cancer Sci. 2013; 104:1362–7.

<https://doi.org/10.1111/cas.12235> PMID:[23859808](https://pubmed.ncbi.nlm.nih.gov/23859808)

85. Beebe-Dimmer JL, Dunn RL, Sarma AV, Montie JE, Cooney KA. Features of the metabolic syndrome and prostate cancer in African-American men. Cancer. 2007; 109:875–81.

<https://doi.org/10.1002/cncr.22461> PMID:[17265528](https://pubmed.ncbi.nlm.nih.gov/17265528)

86. Lo SF, Chang SN, Muo CH, Chen SY, Liao FY, Dee SW, Chen PC, Sung FC. Modest increase in risk of specific types of cancer types in type 2 diabetes mellitus patients. Int J Cancer. 2013; 132:182–8.

<https://doi.org/10.1002/ijc.27597> PMID:[22510866](https://pubmed.ncbi.nlm.nih.gov/22510866)

87. Wallström P, Bjartell A, Gullberg B, Olsson H, Wirfält E. A prospective Swedish study on body size, body composition, diabetes, and prostate cancer risk. Br J Cancer. 2009; 100:1799–805.

<https://doi.org/10.1038/sj.bjc.6605077> PMID:[19436298](https://pubmed.ncbi.nlm.nih.gov/19436298)

88. Strom SS, Yamamura Y, Flores-Sandoval FN, Pettaway CA, Lopez DS. Prostate cancer in Mexican-Americans: identification of risk factors. Prostate. 2008; 68:563–70.

<https://doi.org/10.1002/pros.20713> PMID:[18247399](https://pubmed.ncbi.nlm.nih.gov/18247399)

89. Pourmand G, Salem S, Mehrsai A, Lotfi M, Amirzargar MA, Mazdak H, Roshani A, Kheirollahi A, Kalantar E, Baradaran N, Saboury B, Allameh F, Karami A, et al. The risk factors of prostate cancer: a multicentric case-control study in Iran. Asian Pac J Cancer Prev. 2007; 8:422–8.

PMID:[18159981](https://pubmed.ncbi.nlm.nih.gov/18159981)

90. Yeh HC, Platz EA, Wang NY, Visvanathan K, Helzlsouer KJ, Brancati FL. A prospective study of the associations between treated diabetes and cancer outcomes. Diabetes Care. 2012; 35:113–8.

<https://doi.org/10.2337/dc11-0255> PMID:[22100961](https://pubmed.ncbi.nlm.nih.gov/22100961)

91. Li C, Balluz LS, Ford ES, Okoro CA, Tsai J, Zhao G. Association between diagnosed diabetes and self-reported cancer among U.S. adults: findings from the 2009 Behavioral Risk Factor Surveillance System. Diabetes Care. 2011; 34:1365–8.

<https://doi.org/10.2337/dc11-0020> PMID:[21505205](https://pubmed.ncbi.nlm.nih.gov/21505205)

92. Attner B, Landin-Olsson M, Lithman T, Noreen D, Olsson H. Cancer among patients with diabetes, obesity and abnormal blood lipids: a population-based register study in Sweden. Cancer Causes Control. 2012; 23:769–77.

<https://doi.org/10.1007/s10552-012-9946-5> PMID:[22467266](https://pubmed.ncbi.nlm.nih.gov/22467266)

93. Adami HO, McLaughlin J, Ekbom A, Berne C, Silverman D, Hacker D, Persson I. Cancer risk in patients with diabetes mellitus. Cancer Causes Control. 1991; 2:307–14.

<https://doi.org/10.1007/BF00051670> PMID:[1932543](https://pubmed.ncbi.nlm.nih.gov/1932543)

94. Martin RM, Vatten L, Gunnell D, Romundstad P, Nilsen TI. Components of the metabolic syndrome and risk of prostate cancer: the HUNT 2 cohort, Norway. Cancer Causes Control. 2009; 20:1181–92.

<https://doi.org/10.1007/s10552-009-9319-x> PMID:[19277881](https://pubmed.ncbi.nlm.nih.gov/19277881)

95. Wu C, Moreira DM, Gerber L, Rittmaster RS, Andriole GL, Freedland SJ. Diabetes and prostate cancer risk in the REDUCE trial. Prostate Cancer Prostatic Dis. 2011; 14:326–31.

<https://doi.org/10.1038/pcan.2011.28> PMID:[21709690](https://pubmed.ncbi.nlm.nih.gov/21709690)

96. Bao C, Pedersen NL, Yang R, Marseglia A, Xu W, Wang Y, Qi X, Xu W. Diabetes in midlife and risk of cancer in late life: A nationwide Swedish twin study. Int J Cancer. 2018; 143:793–800.

<https://doi.org/10.1002/ijc.31365> PMID:[29566433](https://pubmed.ncbi.nlm.nih.gov/29566433)

97. Magliano DJ, Davis WA, Shaw JE, Bruce DG, Davis TM. Incidence and predictors of all-cause and site-specific cancer in type 2 diabetes: the Fremantle Diabetes Study. Eur J Endocrinol. 2012; 167:589–99.

<https://doi.org/10.1530/EJE-12-0053> PMID:[22893694](https://pubmed.ncbi.nlm.nih.gov/22893694)

98. Guerrios-Rivera L, Howard LE, Wiggins EK, Hoyo C, Grant DJ, Erickson TR, Ithisuphalap J, Freedland AR, Vidal AC, Fowke JH, Freedland SJ. Metabolic syndrome is associated with aggressive prostate cancer regardless of race. Cancer Causes Control. 2023; 34:213–21.

<https://doi.org/10.1007/s10552-022-01649-9> PMID:[36450931](https://pubmed.ncbi.nlm.nih.gov/36450931)

99. González-Pérez A, García Rodríguez LA. Prostate cancer risk among men with diabetes mellitus (Spain). Cancer Causes Control. 2005; 16:1055–8.

<https://doi.org/10.1007/s10552-005-4705-5> PMID:[16184470](https://pubmed.ncbi.nlm.nih.gov/16184470)

100. Wallner LP, Morgenstern H, McGree ME, Jacobson DJ, St Sauver JL, Jacobsen SJ, Sarma AV. The effects of metabolic conditions on prostate cancer incidence over 15 years of follow-up: results from the Olmsted County Study. BJU Int. 2011; 107:929–35.

<https://doi.org/10.1111/j.1464-410X.2010.09703.x> PMID:[20880183](https://pubmed.ncbi.nlm.nih.gov/20880183)

101. Carstensen B, Read SH, Friis S, Sund R, Keskimäki I, Svensson AM, Ljung R, Wild SH, Kerssens JJ, Harding JL, Magliano DJ, Gudbjörnsdottir S, and Diabetes and Cancer Research Consortium. Cancer incidence in persons with type 1 diabetes: a five-country study of 9,000 cancers in type 1 diabetic individuals. Diabetologia. 2016; 59:980–8.

<https://doi.org/10.1007/s00125-016-3884-9> PMID:[26924393](https://pubmed.ncbi.nlm.nih.gov/26924393)

102. Bjornsdottir HH, Franzén S, Rawshani A. Cancer incidence and mortality among 457,473 persons with type 2 diabetes compared to 2,287,365 matched controls in Sweden: An observational study. in Diabetologia vol. vol.61 S591. 2018.

103. Karlin NJ, Kosiorek HE, Verona PM, Coppola KE, Cook CB. Cancer, diabetes, survival and glycemic control: a large multisite analysis. Future Sci OA. 2023; 8:FSO820.

<https://doi.org/10.2144/fsoa-2022-0018> PMID:[36788982](https://pubmed.ncbi.nlm.nih.gov/36788982)

104. Hu Y, Zhang X, Ma Y, Yuan C, Wang M, Wu K, Tabung FK, Tobias D, Hu FB, Giovannucci E, Song M. Incident Type 2 Diabetes Duration and Cancer Risk: A Prospective Study in Two US Cohorts. J Natl Cancer Inst. 2021; 113:381–9.

<https://doi.org/10.1093/jnci/djaa141>
PMID:[33225344](https://pubmed.ncbi.nlm.nih.gov/33225344)

105. Chen CB, Eurich DT, Majumdar SR, Johnson JA. Risk of prostate cancer across different racial/ethnic groups in men with diabetes: a retrospective cohort study. Diabet Med. 2018; 35:107–11.

<https://doi.org/10.1111/dme.13536> PMID:[29078006](https://pubmed.ncbi.nlm.nih.gov/29078006)

106. Walker JJ, Brewster DH, Colhoun HM, Fischbacher CM, Leese GP, Lindsay RS, McKnight JA, Philip S, Sattar N, Stockton DL, Wild SH, and Scottish Diabetes Research Network (SDRN) Epidemiology Group. Type 2 diabetes, socioeconomic status and risk of cancer in Scotland 2001-2007. Diabetologia. 2013; 56:1712–5.

<https://doi.org/10.1007/s00125-013-2937-6> PMID:[23661106](https://pubmed.ncbi.nlm.nih.gov/23661106)
